# Unveiling Intercalation Chemistry via Interference‐Free Characterization Toward Advanced Aqueous Zinc/Vanadium Pentoxide Batteries

**DOI:** 10.1002/advs.202405134

**Published:** 2024-08-29

**Authors:** Xianjin Li, Yue Xu, Xiaoqin Chen, Xiaofei Yang, Guohui Zhang, Xianfeng Li, Qiang Fu

**Affiliations:** ^1^ Department of Chemical Physics University of Science and Technology of China Hefei 230026 China; ^2^ State Key Laboratory of Catalysis Dalian Institute of Chemical Physics Chinese Academy of Sciences Dalian 116023 China; ^3^ Division of Energy Storage Dalian National Laboratory for Clean Energy Dalian Institute of Chemical Physics Chinese Academy of Sciences Dalian 116023 China

**Keywords:** energy storage, intercalation mechanisms, interference‐free characterization, V_2_O_5_ cathode, zinc‐ion batteries

## Abstract

Aqueous Zn/V_2_O_5_ batteries are featured for high safety, low cost, and environmental compatibility. However, complex electrode components in real batteries impede the fundamental understanding of phase transition processes and intercalation chemistry. Here, model batteries based on V_2_O_5_ film electrodes which show similar electrochemical behaviors as the real ones are built. Advanced surface science characterizations of the film electrodes allow to identify intercalation trajectories of Zn^2+^, H_2_O, and H^+^ during V_2_O_5_ phase transition processes. Protons serve as the vanguard of intercalated species, facilitating the subsequent intercalation of Zn^2+^ and H_2_O. The increase of capacity in the activation process is mainly due to the transition from V_2_O_5_ to more active V_2_O_5_·nH_2_O structure caused by the partial irreversible deintercalation of H_2_O rather than the increase of active sites induced by the grain refinement of electrode materials. Eventually, accumulation of Zn species within the oxide electrode results in the formation of inactive (Zn_3_(OH)_2_V_2_O_7_·2H_2_O) structure. The established intercalation chemistry helps to design high‐performance electrode materials.

## Graphical abstract

The disputed charge storage mechanism of V_2_O_5_ cathode, caused by interference from complex components in real batteries, is illustrated through advanced surface characterization of film electrode. The phase transition from V_2_O_5_ to V_2_O_5_·nH_2_O proceeds via preferential intercalation of protons into the interlayer, followed by H_2_O and Zn^2+^, and V_2_O_5_ ultimately evolves to Zn_3_(OH)_2_V_2_O_7_·2H_2_O due to Zn^2+^ accumulation in the interlayer.

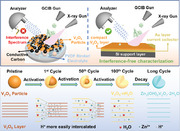

## 1 Introduction

Aqueous zinc ion batteries (ZIBs) are promising candidates for future large‐scale energy storage technologies due to their cost‐effectiveness, high safety, competitive energy density, and environmental friendliness.^[^ ^1^ ^]^ The energy density and power density of ZIBs mainly depend on cathode materials. Many excellent researches ^[^ ^2a–f^ ^]^ and review articles ^[^ ^2g–i^ ^]^ have been devoted to improving the activity and stability of cathode materials. Among various cathode materials, V_2_O_5_ is particularly attractive due to its high theoretical specific capacity (589 mA h g^−1^), easy preparation, and flexible multivalent state of V element.^[^ ^3^ ^]^ Commercial V_2_O_5_ with a layered structure has been often used as the cathode for ZIBs.^[^ ^4^ ^]^ However, the low stability and slow ion migration from the narrow interlayer distance (≈4.4 Å) limit the battery performance.^[^ ^5a–d^ ^]^ Increasing the interlayer spacing is an effective way to improve ion diffusion dynamics.^[^ ^5e^ ^]^ A large number of modified materials have been reported, which show improved performance compared to pristine V_2_O_5_.^[^ ^6^ ^]^ For example, Liu's recent work achieved a high specific capacity of 553 mA h g^−1^ at 0.1 A g^−1^.^[^ ^13c^ ^]^ However, most studies focus on the performance improvement by the modification of pristine V_2_O_5_. Although various charge storage mechanisms of the modified materials have been presented, it is far from a consistent understanding for the battery system.^[^ ^11^, ^12^, ^13^, ^14^, ^15^, ^16^ ^]^ For example, the sequence, proportion, and capacity contribution of each intercalation species (Zn^2+^, H_2_O, and H^+^) needs to be explored.

In some early works, the charge storage mechanism has been simply attributed to the (de)intercalation of Zn^2+^ between the V_2_O_5_ layers, showing the mutual transition between V_2_O_5_ and Zn*_x_* V_2_O_5_ during the charge and discharge processes.^[^ ^4^, ^7^ ^]^ Subsequently, researchers noted that the battery performance always changes significantly with the cycle number. This indicates the more complex mechanisms during battery operation. The capacity increase has often been observed in the early stages of battery cycling, and it is usually attributed to the activation of batteries.^[^ ^8^ ^]^ For example, Zhang et al. observed that pristine V_2_O_5_ particles progressively evolve into porous nanosheets during cycling, supplying more active sites for Zn^2+^ storage and resulting in the initial capacity increase.^[^ ^4a^ ^]^ Meanwhile, the capacity decay always occurs after long‐term cycling due to vanadium dissolution ^[^ ^8^, ^9^ ^]^ and layered structure collapse.^[^ ^10^ ^]^

Among all, it is most important to understand the electrochemical behavior of various intercalated species during charge and discharge processes. Some studies have revealed the important role of H_2_O molecules in metal ion intercalation.^[^ ^11^ ^]^ For example, Nazar et al. reported a high‐performance Zn_0.25_V_2_O_5_·nH_2_O cathode material (a capacity up to 260 mA h g^−1^, and a capacity retention of more than 80% over 1000 cycles). They confirmed that interlayer H_2_O molecules can improve the diffusion kinetics of Zn^2+^.^[^ ^12^ ^]^ Yan et al. demonstrated that H_2_O molecules shield the charge of Zn^2+^ and thus reduce their electrostatic interactions with the V_2_O_5_ framework, effectively promoting their diffusion.^[^ ^12^ ^]^ Chen et al. confirmed that the bound H_2_O can promote the migration of Zn^2+^ by reducing the diffusion energy barrier.^[^ ^2^ ^]^ However, there are controversies over the form in which H_2_O molecules enter the interlayer and how they affect the phase transition of V_2_O_5_ during cycling. One view is that H_2_O molecules coupled with Zn^2+^ in the form of [Zn(H_2_O)_6_]^2+^ are (de)intercalated during the charge and discharge processes.^[^ ^9^, ^13^ ^]^ On the contrary, Deng et al. suggested that H_2_O molecules are not co(de)intercalated with Zn^2+^.^[^ ^14^ ^]^ In addition, Huang and coworkers demonstrated that Zn^2+^ intercalation is accompanied by H_2_O deintercalation upon discharge, while Zn^2+^ deintercalation is coupled with H_2_O intercalation for charge.^[^ ^12^, ^15^ ^]^ Meanwhile, most of research confirmed that the above‐mentioned (de)intercalation processes of H_2_O and/or Zn^2+^ are closely related to the intercalation of protons.^[^ ^16^ ^]^ Although some literature has reported intercalation of protons and Zn^2+^ in several cathode materials, such as (ɛ)‐MnO_2_^[^ ^16^ ^]^ and VO_2_,^[^ ^16^ ^]^ most of them are based on indirect evidence from by‐products or theoretical calculations. Some works show contradictory results,^[^ ^16^ ^]^ which also requires further investigations in V_2_O_5_.

The controversy concerning the aforementioned mechanisms arises from the fact that characterizations of V_2_O_5_ electrode materials are often interfered by other components. The electrodes in real batteries contain various components, including conductive carbon black, polyvinylidene fluoride (PVDF) binder, and residual electrolyte components adsorbed between V_2_O_5_ particles during cycling (Figure S1, Supporting Information). Thus, it is difficult to obtain direct and accurate experimental evidences of structural and chemical changes within the electrode material particles particularly using surface‐sensitive techniques, such as X−ray photoelectron spectroscopy (XPS) and time of flight secondary ion mass spectrometry (TOF−SIMS). Here, we construct model batteries using film electrodes that present similar electrochemical behaviors as the real ones. The well‐defined film electrode is ideal for surface characterization techniques, such as XPS and TOF−SIMS and for depth profile analysis. Meanwhile, intercalation trajectories of each intercalated species and phase transition processes can be tracked by isotope tracing measurements and quantitative depth profiling. This allows for the accurate description of the capacity contribution of all intercalated species at various stages of the battery cycle. It can be considered as a kind of “interference‐free characterization.” The results show that the activation process proceeds from the surface layer of active material particles to internal transformation. Protons preferentially intercalate into the interlayer during discharging which is followed by deep intercalation of H_2_O and Zn^2+^, and a slow accumulation of H_2_O with the increase of corresponding capacity. The capacity contribution before discharge to 0.6 V mainly comes from protons intercalation, while the discharge capacity in the lower potential range is mainly contributed by Zn^2+^ ions. Besides the increase in the number of active sites caused by grain refining of electrode materials or battery activation,^[^ ^8^ ^]^ the gradually increased capacity at the beginning is mainly attributed to the phase transition from V_2_O_5_ to V_2_O_5_·nH_2_O. The formation of Zn_3_(OH)_2_V_2_O_7_·2H_2_O is due to Zn^2+^ ions accumulation between V_2_O_5_ layers and vanadium dissolution, which leads to capacity attenuation. As a result, the phase transition and intercalation chemistry of V_2_O_5_ electrode materials have been well established, enabling the development of vanadium oxide‐based electrode materials with enhanced battery performance.

## 2 Results and Discussion

### 2.1 Structure and Performance of Commercial V_2_O_5_ Cathode

**Figure 1:**
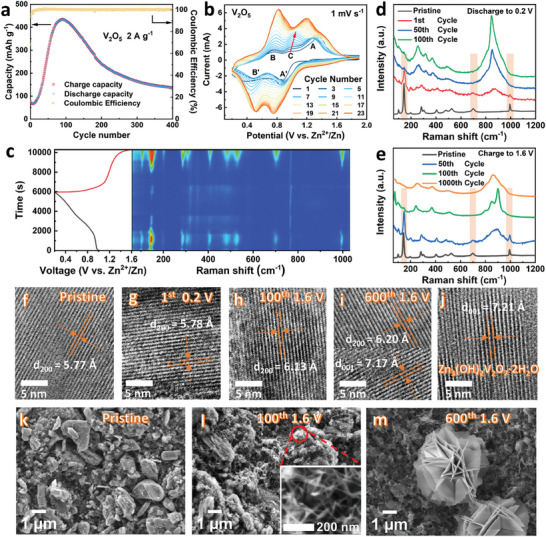
a,b) Electrochemical performance of Zn/V_2_O_5_ real coin cells using commercial V_2_O_5_ cathode material: a) Long‐term cycling performance at 2 A g^−1^. b) Cyclic voltammetry curves at 1 mV s^−1^. c) Operando Raman spectra of the V_2_O_5_ electrode during the first discharge/charge cycle. d,e) Raman spectra of the V_2_O_5_ electrode at different cycle numbers under the discharge d) and charge e) conditions. f−j) HRTEM images showing the lattice spacing of V_2_O_5_ electrode at different states of pristine f), 1st cycle discharge to 0.2 V g), 100th cycle charge to 1.6 V h), 600th cycle charge to 1.6 V i), and prepared Zn_3_(OH)_2_V_2_O_7_·2H_2_O j). k–m) FESEM images of the cathode surface at different states of pristine k), the 100th cycle charge to 1.6 V l), and 600th cycle charge to 1.6 V m).

We first investigate the performance of real coin cells using commercial V_2_O_5_ as the cathode material (Zn plate as anode, 3 mol kg^−1^ Zn(OTf)_2_ in water as electrolyte). **Figure** **1a**,b shows long‐term cycling performance and cyclic voltammetry (CV) test results from the real coin cells. At the beginning of the long cycle (Figure 1a), the battery capacity is only 70 mA h g^−1^ at 2 A g^−1^. Then, it increases continuously until the 100th cycle, reaching a peak capacity of 430 mA h g^−1^. After that, its capacity gradually decreases to 130 mAh g^−1^ at the 400th cycle. For CV curve of the 2nd cycle at 1 mV s^−1^ shown in Figure S2 (Supporting Information), two distinct pairs of redox peaks at 0.90/1.27 V (peak A’/A) and 0.49/0.76 V (peak B’/B) can be ascribed to the valence changes of vanadium from V^5+^ to V^4+^ and V^4+^ to V^3+^, respectively. Figure 1b shows that their peak current intensity increases as the cycle progresses, coinciding with the increase in battery capacity. It is noted that a weak oxidation peak at ≈1.0 V (peak C in Figure 1b) becomes stronger during subsequent cycles. Eventually peak C merges with peak A, which corresponds to the formation of a new phase.

Field emission scanning electron microscopy (FESEM) images of the Zn‐anode after cycling given in Figure S3 (Supporting Information) indeed show an increase in surface roughness. The energy dispersive spectrometer (EDS) mapping images (Figure S4, Supporting Information) and XPS spectra (Figure S5, Supporting Information) indicate that the main component of the by‐products is ZnO. But X−ray diffraction (XRD) results (Figure S6, Supporting Information) show that there are no other miscellaneous peaks except Zn, indicating that the amount of ZnO by‐product is not large and not the main reason for attenuation of the battery capacity. In order to avoid dendrites penetrating the separator and causing battery failure, a thicker GF/D glass fiber separator (675 µm) is used as the separator in this work.

Operando Raman characterization has been conducted to investigate the structural evolution of the V_2_O_5_ cathode during the initial discharging/charging process. As shown in Figure 1c (3D pattern in Figure S7, Supporting Information), a strong band at 148 cm^−1^ is indexed to the skeleton bending vibration of V−O−V bond. The peak at 702 cm^−1^ corresponds to the doubly coordinated oxygen (V_2_−O) stretching mode owing to corner‐shared O common to two pyramids. The band at 996 cm^−1^ is assigned to the in‐phase stretching vibrational mode of the apical V═O bond.^[^ ^17^ ^]^ Upon discharge, the intensities of these peaks gradually diminish followed by their recovery during charging to 1.6 V, which indicates that V_2_O_5_ still retains its original lattice structure after the first discharge−charge cycle. At this stage, the battery reached the cutoff voltage at a significantly low capacity, thereby preserving a relatively intact lattice structure. That is due to the low Zn^2+^ ions diffusion rate and high ohmic polarization caused by the narrow V_2_O_5_ layer spacing.

It should be noted that in the different stages of the long‐term cycle, phase components of the electrode materials may dynamically change. Therefore, we selected the electrode samples presenting various battery capacities (Figure 1a), including the initial cycle (1st cycle), capacity rise (50th cycle), peak capacity (100th cycle), and capacity decline (1000th cycle). Figure 1d,e; and Figure S8 (Supporting Information) show Raman spectra of the V_2_O_5_ cathode in the different cycle stages under both discharge and charge conditions. The orange bands marked at 148, 702, and 996 cm^−1^ represent the characteristic vibration peak of V_2_O_5_. These Raman results confirm that the V_2_O_5_ structure gradually degrades as the battery capacity increases and no V_2_O_5_ crystals are left as seen from the complete disappearance of all V_2_O_5_ Raman signals at the maximum capacity. The V_2_O_5_ electrodes in the above cycle states are further characterized by XRD. As depicted in Figure S9a (Supporting Information), after discharging to 0.2 V in the first cycle the patterns from pristine V_2_O_5_ (PDF#41−1426) are still discernible. At the 50th cycle, the inherent peaks at 15.3° of V_2_O_5_ disappear when the battery is discharged to 0.2 V (Figure S9b, Supporting Information). Nevertheless, upon further charging to 1.6 V standard V_2_O_5_ peaks reappear (Figure S10a, Supporting Information), indicating that activation process has not yet been completed at the 50th cycle.

When the capacity reaches its peak at the 100th cycle, the inherent peaks at 15.3° of V_2_O_5_ could not be detected in either discharge (Figure S9c, Supporting Information) or charge state (Figure S10b, Supporting Information). Meanwhile, overlapping peaks of several possible phases were detected by XRD given in Figures S9–S10 (Supporting Information), such as V_2_O_5_·H_2_O (PDF#21−1432), H_0.39_V_2_O_5_ (PDF#38−0009), and H_1.43_V_2_O_5_ (PDF#40−1114), suggesting that H_2_O molecules and protons may intercalate during discharge. This can be confirmed by thermogravimetry (TG) analysis (Figure S11, Supporting Information) and other characterizations discussed later. It should be noted that the phase of Zn_3_(OH)_2_V_2_O_7_·2H_2_O can be also detected. We also synthesized V_2_O_5_·1.6H_2_O as the electrode material (Figure S12, Supporting Information). The Zn/V_2_O_5_·1.6H_2_O cell performance and its evolution trend of CV curves are consistent with those when Zn/V_2_O_5_ system reaches its capacity peak (Figures S13–S14, Supporting Information). Raman spectra and XRD results also confirm the formation of V_2_O_5_·nH_2_O phases with the increasing capacity and the formation of Zn_3_(OH)_2_V_2_O_7_·2H_2_O phase after the capacity decay (Figures S15−S17, Supporting Information).

High‐resolution transmission electron microscopy (HRTEM) (Figure S18, Supporting Information) was employed to further characterize the phase evolution of V_2_O_5_. Figure 1f shows lattice fringes of the pristine V_2_O_5_ with 5.77 Å lattice spacing corresponding to the (200) plane.^[^ ^4^ ^]^ When the battery was discharged to 0.2 V at the first cycle (Figure 1g), the lattice spacing remains at 5.78 Å due to inadequate reaction. Alternatively, lattice contraction induced by Zn^2+^ intercalation and expansion caused by the H_2_O intercalation may cancel each other out.^[^ ^13^ ^]^ At the 100th cycle (Figure 1h), the layer spacing reached 6.13 Å under a fully charged state, in comparison with the similar result of 6.20 Å at the 600th cycle. The expansion caused by the irreversible deintercalation of H_2_O triggers the transform of V_2_O_5_ to V_2_O_5_·nH_2_O. Meanwhile, at the 600th cycle (Figure 1i), 7.17 Å lattice spacing was also detected, which is consistent with the (001) plane of prepared Zn_3_(OH)_2_V_2_O_7_·2H_2_O in Figure 1j, indicating the generation of Zn_3_(OH)_2_V_2_O_7_·2H_2_O.

FESEM was employed to characterize the morphology during above phase evolution processes (Figure 1k–m). The size of V_2_O_5_ particles does not decrease significantly after 100th cycle, but many sheet structures are generated on the particles surface (inset of Figure 1l; and Figure S19, Supporting Information). When cycling to the 600th cycle, many clusters consisting of sheet structures are formed (Figure 1m), which have the same morphology as Zn_3_(OH)_2_V_2_O_7_·2H_2_O.^[^ ^19^ ^]^ EDS mapping results show that the sheet structure contains O, V, and Zn elements (Figures S20‐S21, Supporting Information), which indicates that phase evolution is accompanied by the formation of attenuation product of Zn_3_(OH)_2_V_2_O_7_·2H_2_O.

### 2.2 Model Battery with V_2_O_5_ Film Electrode

In order to investigate the (de)intercalation behaviors of various species, a V_2_O_5_ film electrode was prepared by magnetron sputtering as illustrated by Figure S22 (Supporting Information). An Au substrate serves as the current collector for the film electrode, thereby ensuring minimal contact resistance between V_2_O_5_ and the current collector. Raman spectrum of the V_2_O_5_ film is consistent with that of commercial V_2_O_5_ powders (Figure S23a, Supporting Information). Thickness of the V_2_O_5_ film electrode measured by atomic force microscope (AFM) is about 170 nm (Figure S24, Supporting Information).

**Figure 2:**
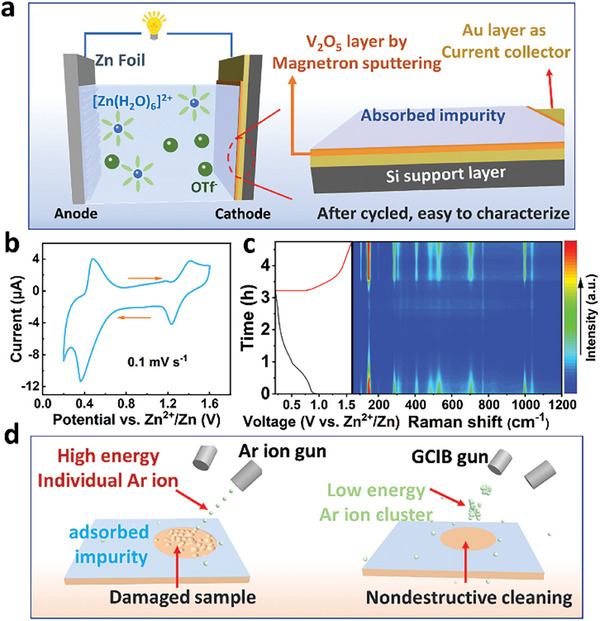
a) Schematic diagram of a model battery construction using a film electrode. b) CV curve of the model battery at 0.1 mV s^−1^. c) Operando Raman spectra of a model battery collected in the first cycle. d) Diagram showing the surface cleaning processes using Ar^+^ ions or Ar ion clusters (Ar*_n_* ^+^).

As shown in **Figure** **2a**, the film electrode was used as the cathode to construct a model battery with Zn anode. CV curve of the model battery at 0.1 mV s^−1^ (Figure 2b) is similar to that of the real battery. Operando Raman spectra during the first cycle are depicted in Figure 2c (with a 3D pattern presented in Figure S23b, Supporting Information). The intensity of the typical V_2_O_5_ signals decreases during discharging and gradually increases during subsequent charging, which is also consistent with those in the real battery (Figure 1c). The CV and Raman data indicate that the model battery presents the same electrochemical behaviors as the real one. Therefore, characterizations over the film electrode can be applied to explore the intercalation chemistry of V_2_O_5_ in ZIBs.

The well‐defined film electrode structure allows precise characterization of interface electrochemical processes as long as its surface is properly cleaned. It is known that Ar^+^ ion sputtering may cause excessive damage to oxide surfaces ^[^ ^18^ ^]^ and thus a gas cluster ion beam (GCIB) has been applied for the surface cleaning. As shown in Figure 2d, each cluster consists of ≈2500 Ar atoms and carries a positive charge. Sample surfaces can be effectively cleaned using GCIB but without much damage (Figure S25, Supporting Information).

### 2.3 Intercalation Trajectories in V_2_O_5_ Activation Process

**Figure 3:**
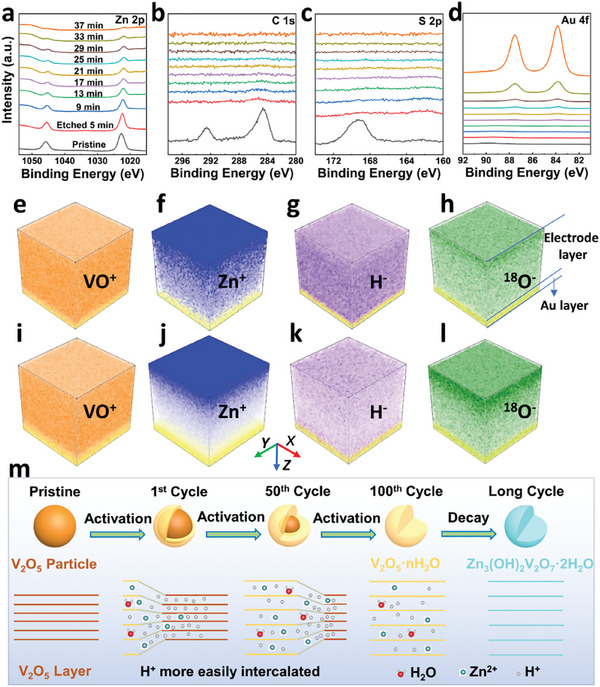
a−d) Depth‐dependent XPS profiles of V_2_O_5_ film electrode discharged to 0.6 V: Zn 2p a), C 1s b), S 2p c), and Au 4f d). e−l) TOF−SIMS analysis results of the V_2_O_5_ film electrode discharged to 0.2 V e−h) and then charged to 1.6 V i−l). m) Schematic diagram of V_2_O_5_ phase transition processes.

**Figure** **3**a−d displays depth‐dependent XPS profiles of the film electrode when the model battery is discharged to 0.6 V. It can be seen that Zn 2p, C 1s, and S 2p signals are all detected before etching, while after the first etching the signals of C 1s and S 2p disappear. Thus, impurities such as electrolyte anions (CF_3_SO_3_^−^) adsorbed on the electrode surface have been completely removed and they do not intercalate into the V_2_O_5_ film. In contrast, Zn 2p signal gradually weakens with increasing etching depth and it disappears upon the emergence of Au 4f signal. This indicates that Zn^2+^ intercalation occurs from the surface to the inside of the V_2_O_5_ film during discharging.

The model V_2_O_5_ electrodes were subsequently analyzed using TOF−SIMS at different charging and discharging states. Here, the electrolyte was prepared using H_2_^18^O as the solvent to track the (de)intercalation of H_2_O molecules. To enhance the signal‐to‐noise ratio, positive ion mode is employed for detecting Zn^2+^, while negative ion mode is utilized for detecting protons and H_2_O molecules. The signal intensity of Zn^+^ secondary ion can reflect the concentration of intercalated Zn^2+^; and H^−^ and ^18^O^−^ represent the number of intercalated protons and H_2_O molecules, respectively. Figure 3e−h shows the TOF−SIMS results when the film electrode was discharged to 0.2 V. It can be seen that both Zn^+^ and ^18^O^−^ are concentrated at the side close to the cathode/electrolyte interface. As the etching depth increases, their concentrations keep decreasing. In contrast, H^−^ signals with an almost uniform concentration distribution are detected throughout the whole V_2_O_5_ film (Figure 3g), indicating that protons can more easily intercalate into V_2_O_5_ layer than Zn^2+^. Thus, protons act as the vanguard for various intercalated species. This result can be further confirmed by subsequent quantitative analysis of XPS data. After the charge to 1.6 V (Figure 3i−l), all the above‐intercalated species are still detectable, demonstrating that they cannot completely de‐intercalate from the V_2_O_5_ layers during charging. Notably, the ^18^O^−^ signal is stronger at the side of the V_2_O_5_ film adjacent to the cathode/electrolyte interface (Figure 3l), suggesting that the phase transition from V_2_O_5_ to V_2_O_5_·nH_2_O happens in the surface layer of the film electrode after the first cycle. Film cathode's long cycle CV curves and capacity evolution are shown in Figure S26 (Supporting Information). TOF−SIMS results of the electrode after 10 discharge and charge cycles are displayed in Figure S27 (Supporting Information). In comparison with the first cycle data depicted in Figure 3e−l, both Zn^+^ and ^18^O^−^ signals are observed to penetrate deeper into the V_2_O_5_ layers and residual H_2_O molecule signal is also deeper after the 10th charge.

Accordingly, the activation of V_2_O_5_ electrode has been schematically illustrated by Figure 3m. The activation process proceeds gradually from the surface to the interior of the V_2_O_5_ particles or films. Protons act as the vanguard and gradually open the space between the neighboring V_2_O_5_ layers, facilitating the subsequent intercalation of H_2_O molecules and Zn^2+^ ions. With more residual H_2_O molecules, the transformation of surface layer of V_2_O_5_ into V_2_O_5_·nH_2_O further facilitates the intercalation of more species and promotes transformation of unfinished electrode regions. As the cycle progresses, H_2_O molecules gradually move inward, explaining the battery capacity increase.

### 2.4 Quantitative Analysis of Intercalation Chemistry

**Figure 4:**
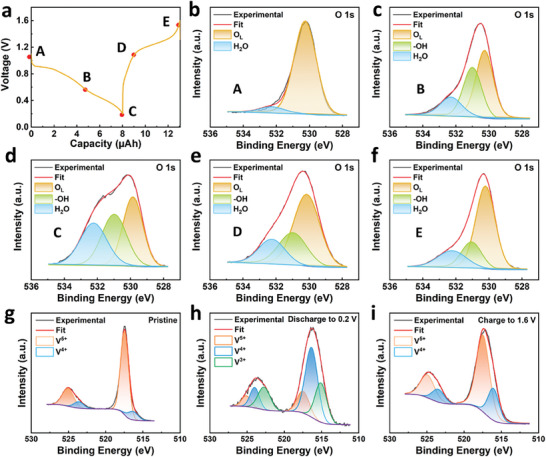
a) Charge and discharge curves of the film electrode. b−f) XPS O 1s spectra of the film electrode at different charge and discharge stages: original uncharged sample b), discharge to 0.6 V c), discharge to 0.2 V d), charge to 1.0 V e), and charge to 1.6 V f). g−i) XPS V 2p spectra of the film electrode at different charge and discharge stages: original uncharged sample g), discharge to 0.2 V h), and charge to 1.6 V i).

XPS has been applied to investigate the model electrodes at the different charge/discharge states as marked in **Figure** **4a**. All model electrode samples were subjected to surface cleaning by GCIB. XPS O 1s spectra were obtained for the pristine model electrode and those discharged to 0.6 and 0.2 V, as well as charged to 1.0 and 1.6 V (Figure 4b−f). O 1s spectra of the pristine model electrode can be resolved into two components from lattice oxygen (O_L_, 530.2 eV) connected to V or Zn and chemically adsorbed water (H_2_O, 532.4 eV).^[^ ^14^, ^20^ ^]^ After cycling in the electrolyte (Figure 4c−f), a new peak at 531.0 eV is attributed to hydroxyl group (−OH, 531.0 eV) formed by the combination of the intercalated protons with the O_L_. The peak area of H_2_O molecules with a binding energy of 532.4 eV increases as the depth of discharge progresses but gradually decreases during subsequent charging (Figure 4c−f). This indicates that H_2_O molecules which are embedded during discharging are expelled from the layers during charging. Notably, even when the film electrode was charged to 1.6 V residual H_2_O molecules were still detectable (Figure 4f). This observation explains the gradual accumulation of H_2_O molecules during the activation process mentioned earlier, which leads to the transformation of V_2_O_5_ into V_2_O_5_·nH_2_O.

XPS V 2p spectra are shown in Figure 4g–i. In the pristine electrode, most V is in +5 oxidation state, with a small portion in +4 state. When the battery was discharged to 0.2 V, V^5+^ peaks at 517.4/525.0 eV decrease significantly, while V^4+^ (516.3/523.9 eV) peaks increase and V^3+^ (515.1/522.7 eV) peaks appear, indicating that V^5+^ is reduced to V^4+^ and V^3+^.^[^ ^7^, ^19^ ^]^ When charged to 1.6 V, valence states are mainly +4 and +5, indicating that some V atoms are not fully oxidized, which is consistent with the detectable Zn peak in Figure S28d (Supporting Information).

To facilitate a more direct comparison of the proportional relationship between intercalated species, O 1s peak areas at each voltage are provided in Table S1 (Supporting Information). Meanwhile, according to XPS O 1s, V 2p, and Zn 2p spectra at different voltages (Figure S28, Supporting Information), their atomic concentration ratios were listed in Table S2 (Supporting Information) and summarized in Figure S29 (Supporting Information). The above data demonstrate that protons are fully intercalated at 0.6 V during discharging, and their proportions undergo slight changes (from 34.5% to 33.3%) as the discharge continues to 0.2 V. Meanwhile, the proportion of Zn^2+^ increases from 9.2% to 33.0% (see Figure S28 and Tables S1 and S2, Supporting Information). Therefore, the discharge capacity in the high potential range (>0.6 V) is mainly contributed by protons. In the low potential range (0.6–0.2 V), it is primarily contributed by Zn^2+^.

Additionally, when the battery is discharged to 0.2 V, the concentration ratio between co‐intercalated H_2_O molecules and intercalated Zn^2+^ is 0.52, which is much lower than 6.0 for solvated hydrated Zn ions ([Zn(H_2_O)_6_)]^2+^). This indicates that Zn^2+^ ions may be partially desolvated during the initial discharging process. For one aspect, the high full‐desolvation barrier makes Zn^2+^ alone difficult to intercalate into V_2_O_5_ due to the strong coulombic interaction between divalent Zn^2+^ and highly polar H_2_O molecules. Furthermore, the larger size of [Zn(H_2_O)_6_)]^2+^ (≈5.5 Å) ^[^ ^21^ ^]^ than V_2_O_5_ interlayer distance (≈4.4 Å) may also prevent it from intercalating in the form of [Zn(H_2_O)_6_)]^2+^. Figure S30 (Supporting Information) shows the TG curves measured during the first cycle. Based on the weight losses from structural H_2_O (approximately from 350 to 500 °C),^[^ ^2^, ^12^, ^22^ ^]^ we estimated that there are 1.64 and 0.94 structural H_2_O molecules per formula unit of V_2_O_5_ in the discharge and charge states, respectively. These values are lower than 2.22 and 1.05 measured by XPS, because TG reflects the average result of the bulk properties, and there are some unintercalated parts in the interior region.

When the battery is charged from 0.2 to 1.0 V, the proportions of the −OH peak area decrease from 33.3% to 24.1%, while the proportions of the H_2_O peak area decrease from 30.7% to 20.3%. Given the similar ratio between these two changes, it is speculated that protons escape in a hydrated form (H_3_O^+^) during charging at low potential range. During the charging process from 1.0 to 1.6 V, the proportion of −OH peak area decreases from 24.1% to 19.5%, while that of Zn decreases from 20.7% to 5.7%. This implies that the primary charging capacity in the high potential range is attributed to the deintercalation of Zn^2+^.

### 2.5 Mechanism Analysis of Formed Inactive Structure Zn_3_(OH)_2_V_2_O_7_·2H_2_O

**Figure 5:**
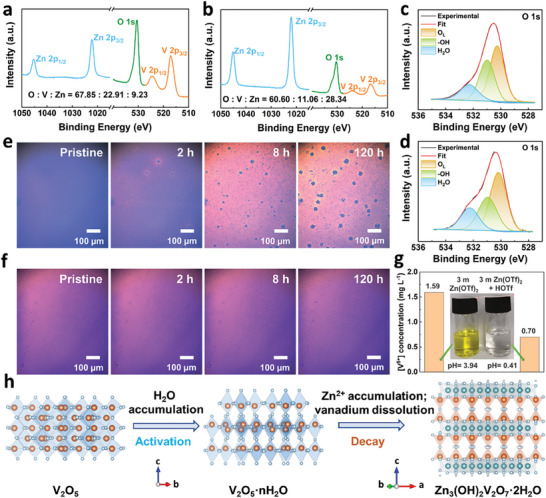
a−d) Comparisons of XPS O, V, and Zn signals and XPS O 1s spectra in the film electrode: discharged to 0.6 V a,c) and soaked for 36 h b,d). e−f) Operando optical microscope photographs of pristine film electrode soaked in e) 3 mol kg^−1^ Zn(OTf)_2_ aqueous solution (pH = 3.94) and f) 3 mol kg^−1^ Zn(OTf)_2_+ HOTf solution (pH = 0.41). g) The concentration of dissolved V in 3 mol kg^−1^ Zn(OTf)_2_ electrolyte with or without HOTf measured by ICP‐AES. Inset: digital photos of the filtrates. h) Schematic diagram of the phase evolution of the V_2_O_5_ cathode during cycling.

During extended cycling, V_2_O_5_ experiences both repetitive (de)intercalation induced by an electric field and constant exposure to the electrolyte, leading to significant capacity decay. To better understand the impacts of (de)intercalation behaviors driven by electric field on the phase transition processes, we conducted two comparative experiments. One film electrode was discharged to 0.6 V, and another was immersed in the electrolyte without voltage for the same time (≈40 min). As expected, the results (**Figure** **5**a,c; and Figure S31 and related explanation of the results in the Supporting Information) suggest that the presence of an electric field promotes the intercalation of Zn^2+^ ions, protons, and H_2_O molecules. However, if the soaking time is extended to 36 h (Figure 5b,d), the amount of intercalated ions exceeds those in the 0.6 V discharge, indicating that prolonged soaking also enhances Zn^2+^ accumulation between V_2_O_5_ layers. Figure S32 (Supporting Information) shows the depth‐dependent XPS results of the film electrode after 50 cycles at 1.6 V (simulating a prolonged cycle state in the real battery), indicating that the cycle process accelerates the accumulation of Zn^2+^ in the interlayer. The irreversibly extracted Zn^2+^ in the interlayer inevitably causes volume change and structural pulverization. The competition between V*─*O and Zn*─*O decreases the structural stability in thermodynamics,^[^ ^23^ ^]^ leading to structure fracture and V_2_O_5_ disollution. Coupled with the strong polarity of H_2_O, the dissolution and the formation of decay products in Zn(OTf)_2_ electrolyte is as follows: ^[^ ^21^, ^24^ ^]^

(1) $$V_{2}O_{5}+3H_{2}O\to 2VO_{2}{{\left(\mathrm{OH}\right)}_{2}}^{-}+2H^{+}$$

(2) $$\begin{array}{c} 2VO_{2}{{\left(\mathrm{OH}\right)}_{2}}^{-}+3Zn^{2+}+3H_{2}O\to Zn_{3}{\left(\mathrm{OH}\right)}_{2}V_{2}O_{7}\cdot 2H_{2}O+4H^{+} \end{array}$$

Taking advantage of the simple composition and flat surface of the film electrode, the formation process of Zn_3_(OH)_2_V_2_O_7_·2H_2_O was observed using in situ optical microscopy (Figure 5e). Initially, the sample surface was flat and smooth. After soaking for 8 h, particles with diameter of about 2 µm formed on the surface. With further extension to 120 h, ≈15 µm particles grew on top of the original foundation. Raman test revealed that these particles were Zn_3_(OH)_2_V_2_O_7_·2H_2_O (Figure S33, Supporting Information). However, after adding HOTf to the electrolyte to achieve a pH of 0.41, no changes were observed even after 120 h (Figure 5f). Inductively coupled plasma‐atomic emission spectrometry (ICP‐AES) measurements also showed that the dissolved V decreased from 1.59 to 0.70 mg L^−1^ after adjusting the pH to 0.41. Thus, HOTf inhibited the dissolution of V_2_O_5_ in the Zn(OTf)_2_ electrolyte and generation of Zn_3_(OH)_2_V_2_O_7_·2H_2_O (Figure 5g; and Tables S3–S5 (Supporting Information) show detailed comparisons of V_2_O_5_ dissolution mechanisms).

The phase transition of V_2_O_5_ during the cycle can be simply illustrated in Figure 5h: the activation process is caused by the accumulation of H_2_O molecules in the layers to transform into V_2_O_5_·nH_2_O, while the accumulation of Zn^2+^ in the interlayers and the dissolution of V_2_O_5_ during prolonged cycling result in the formation of Zn_3_(OH)_2_V_2_O_7_·2H_2_O.

In view of this, shortening activation time and inhibiting Zn_3_(OH)_2_V_2_O_7_·2H_2_O generation can extend the cycling life of the battery. Increasing the concentration of protons in the electrolyte appropriately (the Zn anode unaffected) can not only raise the proportion of protons in the total capacity, thereby reducing Zn^2+^ accumulation between V_2_O_5_ layers and prolonging the duration of peak capacity during cycling (Figure S34, Supporting Information), but also inhibit the formation of Zn_3_(OH)_2_V_2_O_7_·2H_2_O and dissolution of V_2_O_5_. Therefore, H_2_O molecules play the most important role in promoting the phase evolution of the electrode. During the battery cycle, their irreversible deintercalation between layers induces the evolution from V_2_O_5_ to V_2_O_5_·nH_2_O. Their strong polarity promotes the dissolution of V_2_O_5_, leading to the formation of Zn_3_(OH)_2_V_2_O_7_·2H_2_O. The accumulation of Zn^2+^ ions in the interlayer distorts the structure, making it prone to produce degradation products. Protons in Zn(OTf)₂ electrolyte can prevent the dissolution of V₂O₅ and inhibit the formation of Zn_3_(OH)_2_V_2_O_7_·2H_2_O. Therefore, they are not favorable for the phase evolution of the V₂O₅ electrode.

## 3 Conclusion

The model battery has been constructed using a simple and uniform film electrode. After cleaning the surface, the accurate measurements of the electrode were realized via the “interference‐free characterization.” It has been revealed that V_2_O_5_ undergoes a phase transition from V_2_O_5_ to V_2_O_5_·nH_2_O accompanied by an increase in capacity from 70 to 430 mA h g^−1^. It then evolves into attenuation product Zn_3_(OH)_2_V_2_O_7_·2H_2_O, causing the capacity to decrease to 130 mA h g^−1^ after the 400th cycle. The results show that the activation process proceeds from the surface layer of active material particles to internal transformation. Protons preferentially intercalate into the interlayer during discharging which is followed by deep intercalation of H_2_O and Zn^2+^, and a slow accumulation of H_2_O with the increase of corresponding capacity. The discharge capacity in the high potential range is mainly contributed by protons, while it is primarily contributed by Zn^2+^ in the low potential range. The formation of inactive Zn_3_(OH)_2_V_2_O_7_·2H_2_O structure is due to the accumulation of Zn^2+^ in the interlayer during charging and dissolution/deposition of V_2_O_5_. The research methodology employed in this study can serve as a valuable reference for the study of related mechanisms.

## 4 Experimental Section

### Synthesis of the Hydrated V_2_O_5_ Hydrogel (V_2_O_5_·1.6H_2_O)

The hydrated V_2_O_5_ hydrogel was fabricated using a facile hydrothermal method. In brief, 0.72 g of V_2_O_5_ powder was dissolved in a mixture solution containing 60 mL of deionized water and 10 mL of H_2_O_2_ (30 wt%) to obtain a homogeneous clear orange solution. Placing them in a 100 mL Teflon‐lined stainless‐steel autoclave and heating at 190 °C for 10 h to produce the hydrated V_2_O_5_ hydrogel. Pure V_2_O_5_·1.6H_2_O was prepared by freeze‐drying the hydrogel for 48 h.

### Synthesis of the Zn_3_(OH)_2_V_2_O_7_·2H_2_O

1.53 g NaOH was dissolved in 30 mL water, 2.3 g V_2_O_5_ was added into a beaker, and heated with a constant temperature magnetic stirrer. The solution in the cup should be stirred until it becomes pale yellow. ≈1–2 mL of 1, 3−propylenediamine should be added before adding 5.5 g of zinc acetate. Combine the aforesaid solution with a solution made in 21 mL of water. Finally, they were stored in a hydrothermal reactor at 170 °C for 9 h, Zn_3_(OH)_2_V_2_O_7_·2H_2_O nanosheets were obtained.

### Fabrication of V_2_O_5_ Film Electrodes

The V_2_O_5_ film electrodes were prepared by magnetron sputtering with vanadium metal as the target material on the base of gold‐plated silicon wafer. The specific steps are as follows: the gold‐plated silicon wafers were fixed on the sample table, and the metal vanadium target was placed in the magnetron sputtering vacuum chamber. The vacuum degree was 1.0 × 10^−3^ Pa, and then the sample plate temperature was raised to 395 °C at the heating rate of 10 °C per minute, and maintained for 10 min. At this time, the sample plate rotation speed was 10 cycles per minute. The Ar flow rate was adjusted to 90 sccm, O_2_ flow rate to 10 sccm, pressure control vacuum degree to 3 Pa. The sputtering power was maintained at 100 W for 3 min with the sample baffle closed in order to remove contaminants from the surface of the target. Maintaining the aforementioned inlet air flow, adjusting the vacuum level to 2 Pa, setting the sputter power at 200 W, configuring the bias voltage to 50 V, opening the sample baffle, conducting sputtering for a duration of 60 min under these conditions, subsequently cooling down to room temperature, and finally retrieving the film electrodes for further utilization.

### Statistical Analysis

XPS peak fitting analysis was performed using the MultiPak software with a Shirley background and 85/15 Gaussian−Lorentzian fits. The integral intensity of these spectra and the sensitivity factors in the MultiPak software were used for the quantitative evaluation.

## Conflict of Interest

The authors declare no conflict of interest.

## Supporting information

Supporting Information

## Data Availability

The data that support the findings of this study are available from the corresponding author upon reasonable request.
