# Supplementary material for: Unveiling Intercalation Chemistry via Interference‐Free Characterization Toward Advanced Aqueous Zinc/Vanadium Pentoxide Batteries
Source: Adv Sci (Weinh). 2024 Aug 29;11(40):2405134. doi: 10.1002/advs.202405134 (PMC11516048; doi:10.1002/advs.202405134)
Supplement: Supplementary file 1 — Supporting Information [file ADVS-11-2405134-s001.docx]

Supporting Information

Unveiling Intercalation Chemistry via Interference-Free Characterization Toward Advanced Aqueous Zinc/Vanadium Pentoxide Batteries

Xianjin Li, Yue Xu, Xiaoqin Chen, Xiaofei Yang, Guohui Zhang, Xianfeng Li* and Qiang Fu*

**Methods**

**Materials**

High-purity vanadium target material (99.99%) was purchased from Zhongnuo New Material (Beijing) Technology Co., LTD. The gold-plated silicon wafer was purchased from Shuobo super crystalline materials sales center. Commercial V_2_O_5_, Zn(CF_3_SO_3_)_2_, and H_2_^18^O (10 atom%) were all purchased from Macklin Biochemical Technology Co., LTD.

**Electrochemical measurements**

The electrochemical behavior of as−prepared cathode materials (V_2_O_5_, V_2_O_5_·1.6H_2_O, Zn_3_(OH)_2_V_2_O_7_·2H_2_O) were investigated in CR2016−type coin cells. The active materials were mixed with carbon black (Super P) and polyvinylidene difluoride (PVDF) (weight rate 7:2:1) in N−methyl pyrrolidone (NMP). The slurry was cast on stainless steel mesh and directly dried in a vacuum oven at 80 °C for 12 hours. The positive electrodes were cut into circular pieces with a diameter of 14 mm. The loading density was about 2.2± 0.2 mg cm^−2^. The coin cells with an aqueous Zn(OTf)_2_ system were assembled in the atmosphere. The zinc metal foil with a diameter of 14 mm was used as the counter electrode, and the glass fiber membrane (Whatman GF/D) was used as the separator. The 3 mol kg^−1^ zinc trifluoromethane sulfonate (Zn(OTf)_2_) aqueous solution was used as an electrolyte. Galvanostatic charge/discharge tests were performed using the LAND battery test system (Wuhan, China) and NEWARE battery test system with voltages arranged from 0.2− 1.6 V. To investigate the cycling performance of pure V_2_O_5_ and V_2_O_5_·1.6H_2_O, the cells were cycled at 2 A g^−1^. CV measurements were performed on CHI and Biologic VMP3 electrochemical workstation with a scan rate of 1 mV s^−1^ in the voltage range of 0.2− 2.0 V.

**Characterizations**

XRD patterns were recorded on a powder X−ray diffractometer (Rigaku Ultima IV) with Cu Ka radiation at λ = 1.5418 Å. Operando Raman characterizations were performed using a custom−made reaction cell. Raman spectra were recorded with a LabRAM HR 800 Raman spectrometer using a 532 nm laser and a 50x objective. Depth profiling of the surface and interfaces were characterized using TOF−SIMS (IONTOF GmbH, M6, Germany) and XPS (PHI VersaProbe IV, ULVAC−PHI, Japan). The XPS is equipped with a micro−area monochromatized Al Kα radiation (1486.6 eV) with the spot size ranging from 7.5 to 200 μm. The diameter of the X−ray beam in the present work was chosen to be 100 μm. The photoelectron take−off angle was set at 45° and the pass energy was set at 112 eV for the narrow scan under ultrahigh vacuum (UHV) condition (pressure: 3~5 × 10^−9^ mbar). Atomic force microscopy (AFM) characterization was conducted in tapping mode on a Cypher ES AFM (Asylum Research, Oxford Instruments, USA) to obtain the thickness of the V_2_O_5_ film. Operando optical microscope (OM) measurements were performed using the lens that comes with AFM. Field-emission scanning electron microscopy (FE-SEM, JSM-7900F) and transmission electron microscopy (TECNAI G2 F30) were used to investigate the morphology of the as-prepared samples. Energy-dispersive X-ray spectra (EDS) were recorded using an Oxford EDS IE250 system. TG analysis was performed on a thermal analyzer (STA 449 F3, NETZSCH) at a heating rate of 10 ^o^C min^-1^ under the Ar atmosphere.

**Etching parameters optimizing for XPS measurements**

In order to simulate the adsorption of impurities on the electrode surface due to immersion in the electrolyte during cycling, we soaked the pristine uncharged film electrode into the Zn(CF_3_SO_3_)_2_ electrolyte, then took it out after 5 minutes, cleaned its surface electrolyte with deionized water, and dried the surface water with N_2_. Finally, XPS test parameter optimization was conducted under different etching energy and time of GCIB. As a result, the most proper etching parameter is 5 kV, 2.5 minutes each time. Under these conditions, not only can the residual electrolyte adsorbed on the electrode surface be cleaned well but also argon ion clusters will not cause damage to the inorganic layer.


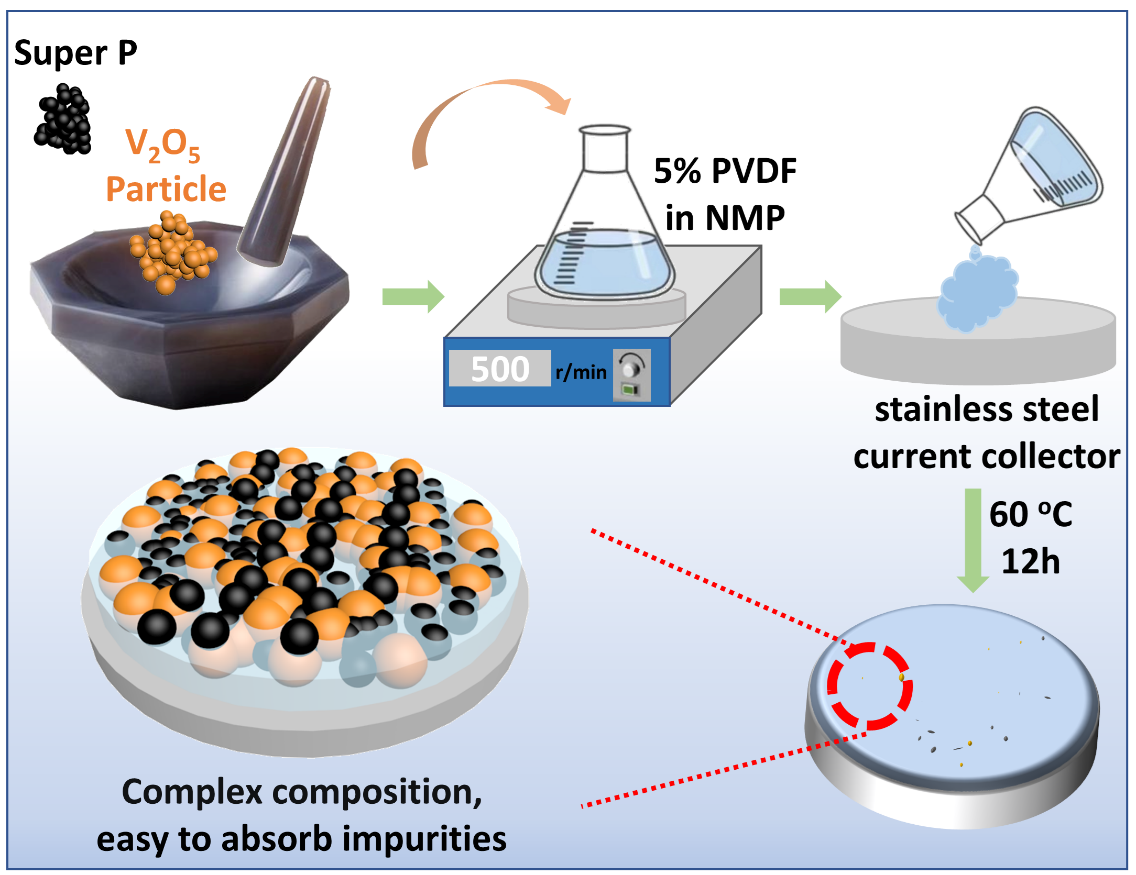


**Figure S1.** Schematic diagram of electrode preparation processes of real coin cell.

Traditional coin-type electrodes contain conductive carbon, binders, and other components. Moreover, the bulk particles of active materials are prone to adsorb solvent molecules, solute ions, etc. Thus, the test results are easily interfered with by these contaminants, which is also one of the important reasons why the charge storage mechanisms are controversial.


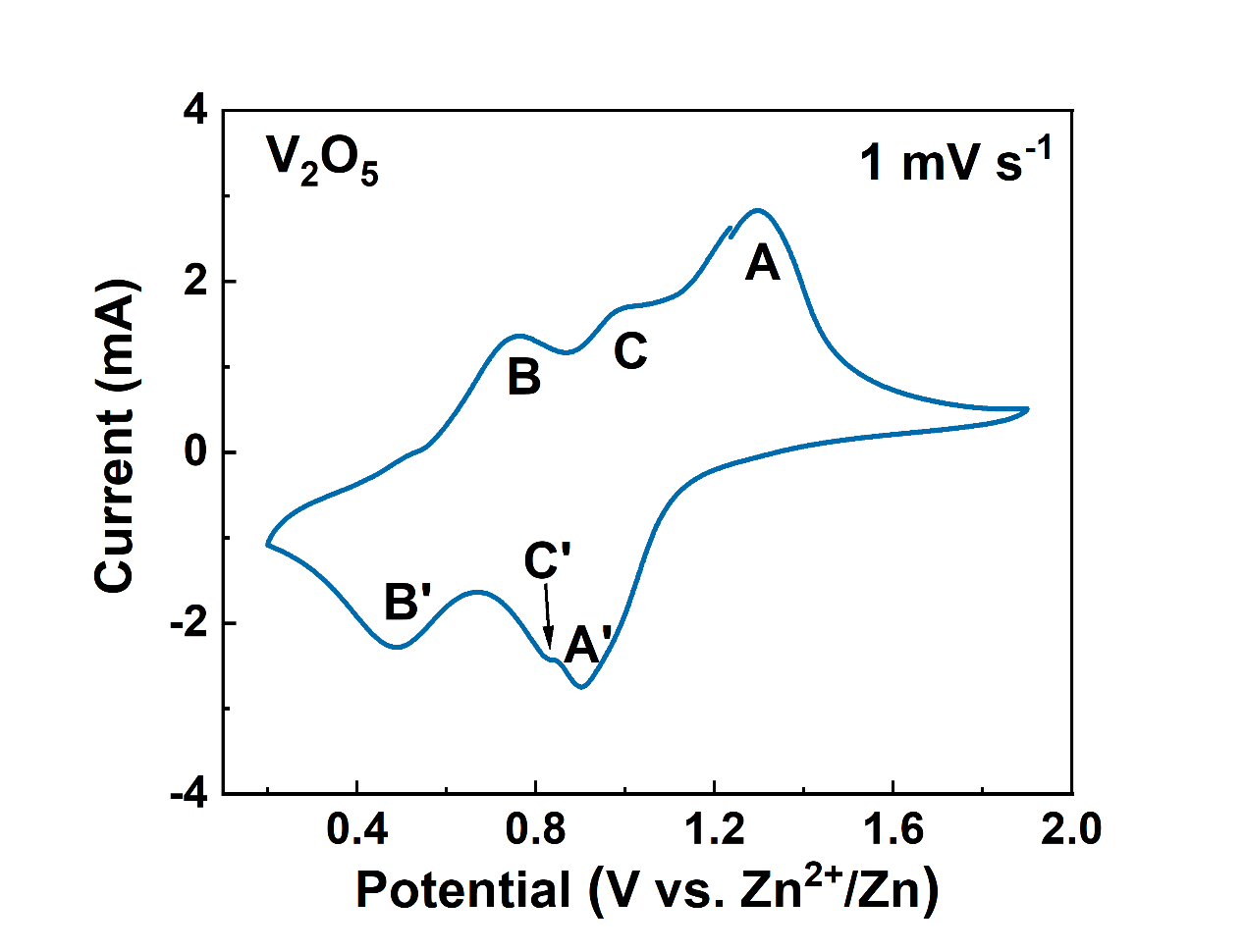


**Figure S2.** Zn/V_2_O_5_ real coin cells’ CV curve of 2^nd^ cycle at 1 mV s^-1^.


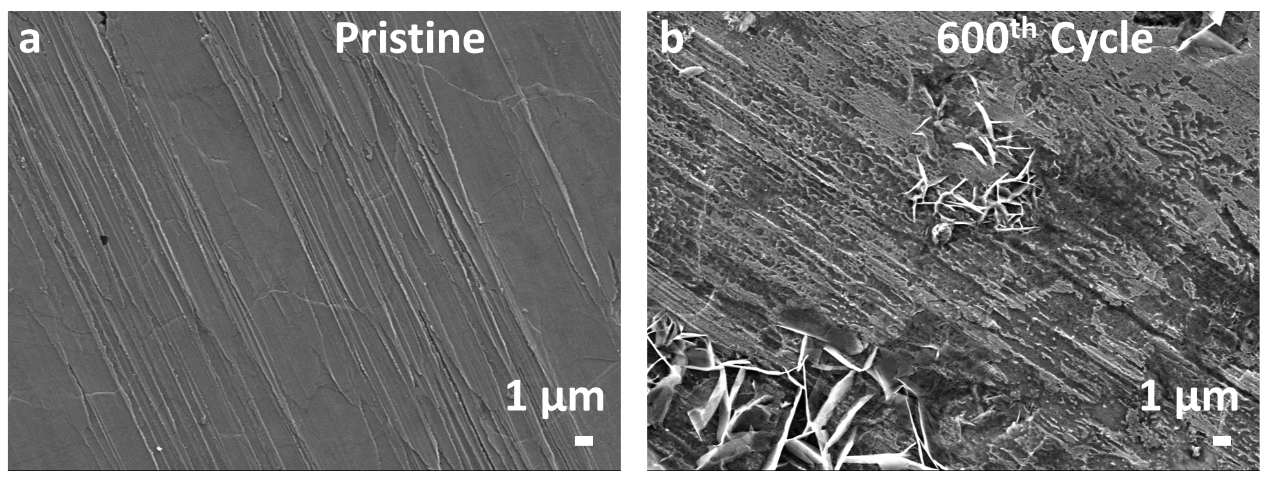


**Figure S3.** SEM images of (a) the pristine Zn anode and (b) the Zn anode after 600 cycles at 2 A g^-1^.


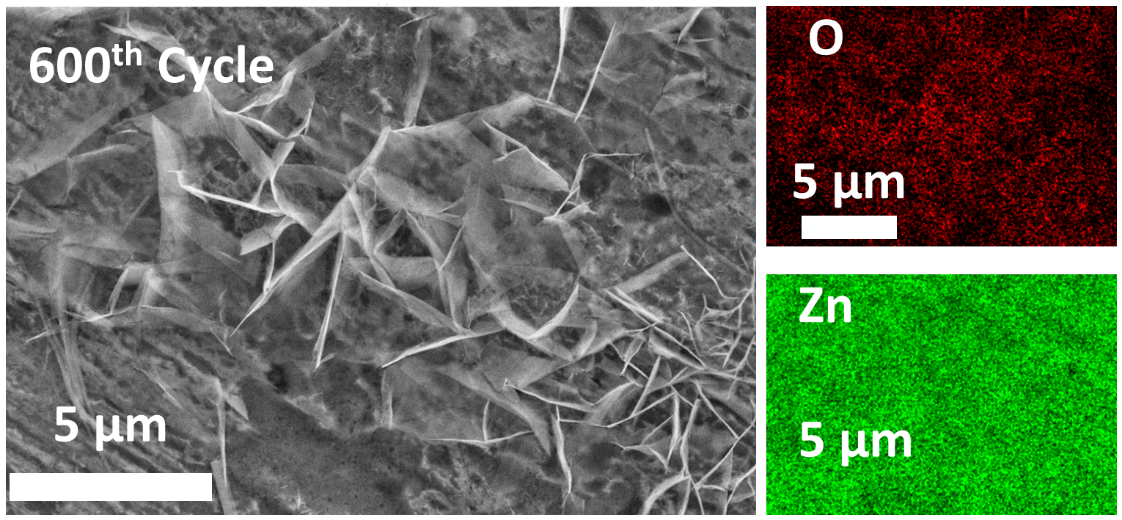


**Figure S4.** EDS mapping images of the Zn anode after 600 cycles at 2 A g^-1^.

**
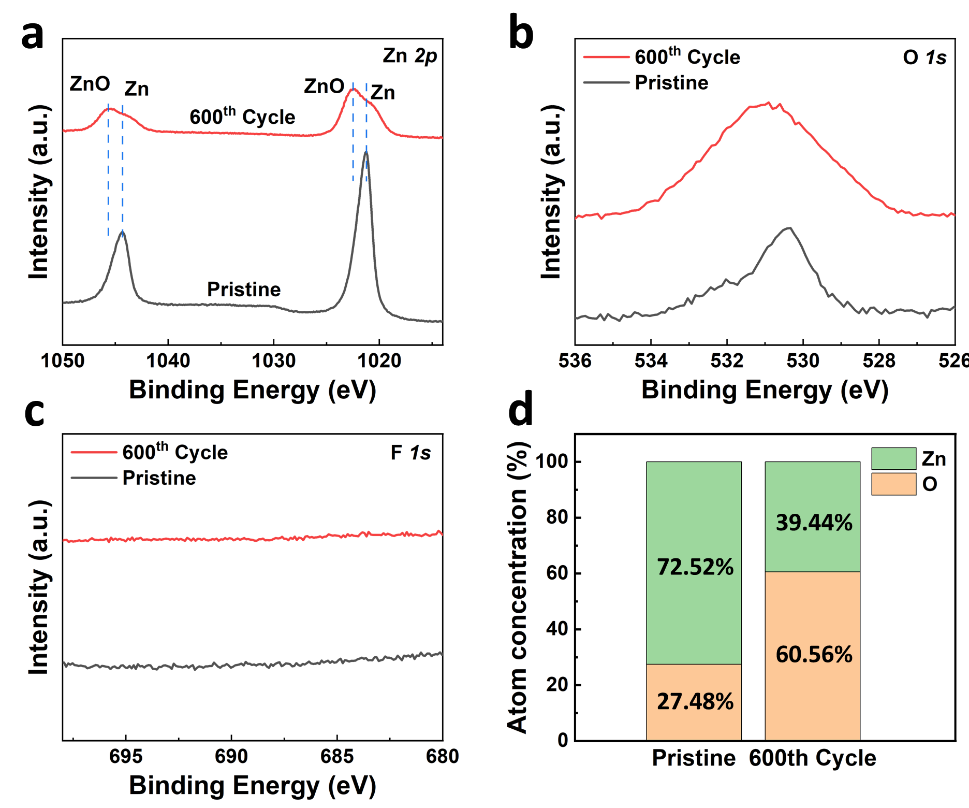
**

**Figure S5.** XPS of the pristine Zn anode and after cycling 600 cycles at 2 A g^-1^. (a) Zn 2p, (b) O 1s, (c) F 1s, and (d) surface atomic concentration ratio of Zn and O.

Compared with the pristine sample, the content of O on the surface of the zinc negative electrode after cycling is greatly increased, but the signal of F 1s is not detected, indicating that the main by-product is ZnO.


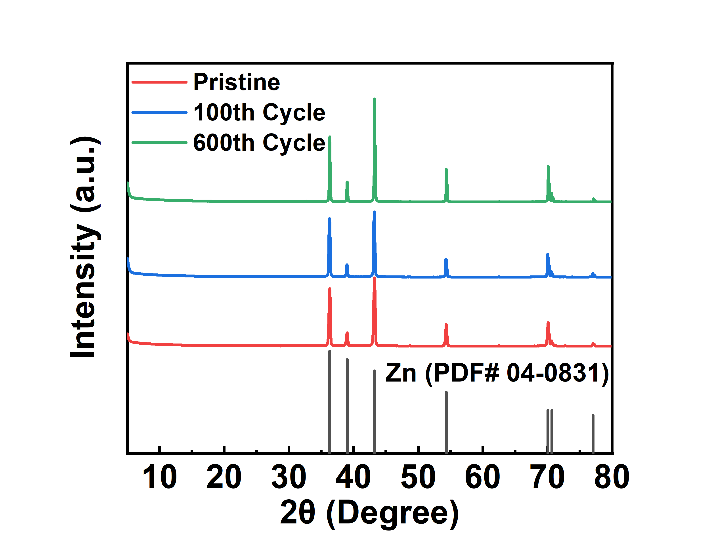


**Figure S6.** XRD patterns of the pristine and cycled Zn anodes compared with the standard PDF cards of Zn.

**
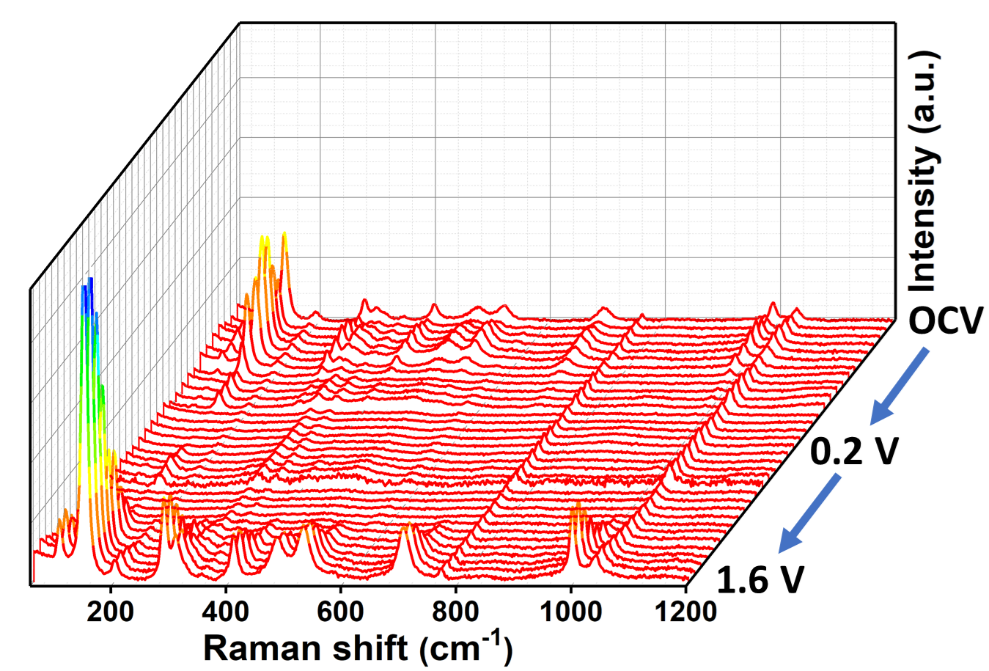
**

**Figure S7.** Operando Raman spectra of the first discharging/charging cycle of Zn/V_2_O_5_ real coin cell.

The peaks at 102 and 200 cm^-1^ are attributed to the bending vibrations of V=O bonds, and the peak at 286 cm^-1^ is assigned to the bending vibrations of O_3_-V-O (B_2g_ mode). Peaks at 408 (V-O_3_-V, A_g_ mode) and 485 cm^-1^ (V-O_2_-V, doubly coordinated oxygen) are consistent with the bending vibrations of the bridging V-O-V. The band at 529 cm^-1^ is assigned to the triply coordinated oxygen (V_3_-O) stretching mode of the edge-shared O in common with three pyramids. The band at 702 cm^-1^ corresponds with the doubly coordinated oxygen (V_2_-O) stretching mode owing to corner-shared O common to two pyramids. The band at 996 cm^-1^ is indexed to the in-phase stretching vibrational mode of the apical V=O bond. ^[1]^


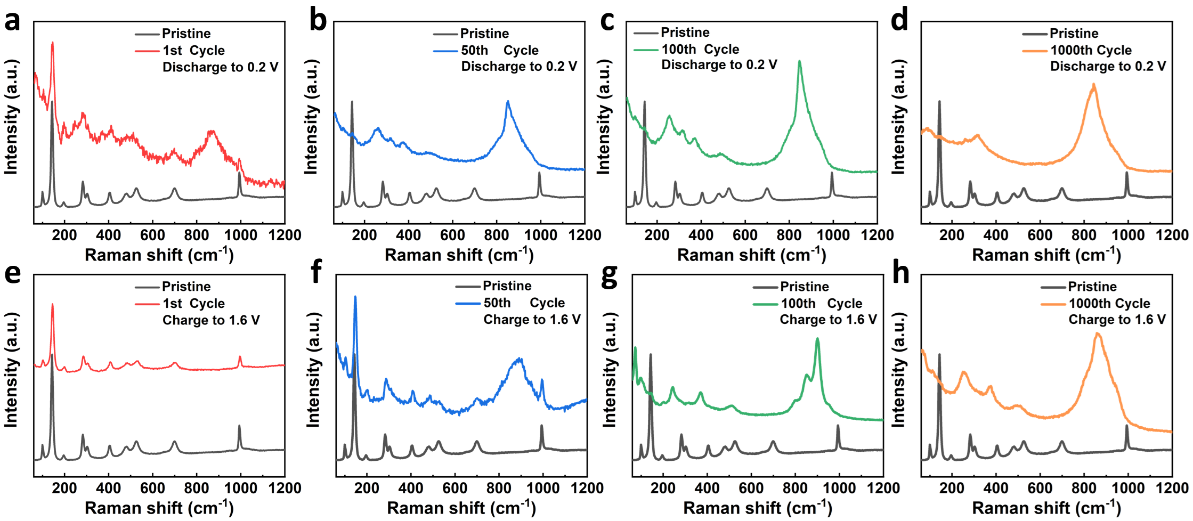


**Figure S8.** Raman spectra of V_2_O_5_ coin-type electrodes at different cycle numbers at (a-d) discharge and (e-h) charge states.

Although the peak shapes are not as sharp as that of the pristine electrode, all inherent peaks of V_2_O_5_ are present when the battery was discharged to 0.2 V during the first cycle (Figure S8a), indicating that intercalated species have no significant destructive effect on the crystal structure of V_2_O_5_ at this time. During the capacity increasing stage, upon full discharging of the battery at the 50^th^ cycle (Figure S8b), the characteristic Raman vibration peaks of V_2_O_5_ at 148, 702, and 996 cm^-1^ are not detectable. This indicates that more V_2_O_5_ is involved in the Zn^2+^ ions intercalation reaction compared to the first cycle, which is consistent with the observed capacity increase in Figure 1a. Upon recharging to 1.6V (Figure S8f) at this point (the 50^th^ cycle), the characteristic peaks of V_2_O_5_ reappear. However, the spectra after the 100^th^ cycle (Figure S8c, g) were consistent with that of the long cycle (Figure S8d, h). No typical peaks of V_2_O_5_ can be detected in either the charge or discharge states. The Raman experiments confirmed that as the capacity increases of V_2_O_5_, its crystal structure progressively diminishes until no intact V_2_O_5_ crystals remain once it reaches its maximum capacity. This is also consistent with the formation of a new peak in the Figure 1b.


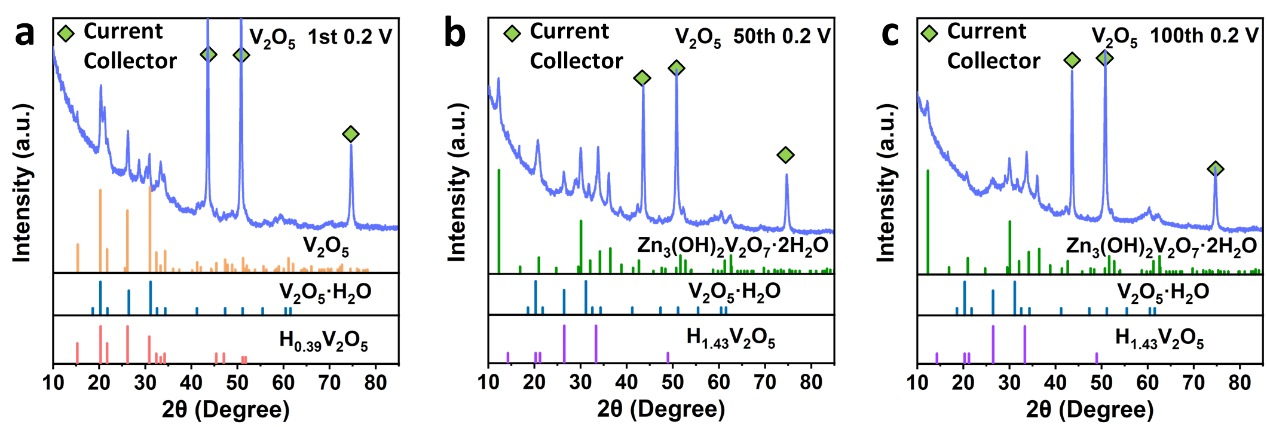


**Figure S9**. XRD patterns of V_2_O_5_ electrode at discharge state of 1^st^ cycle (a), 50^th^ cycle (b), and 100^th^ cycle (c).


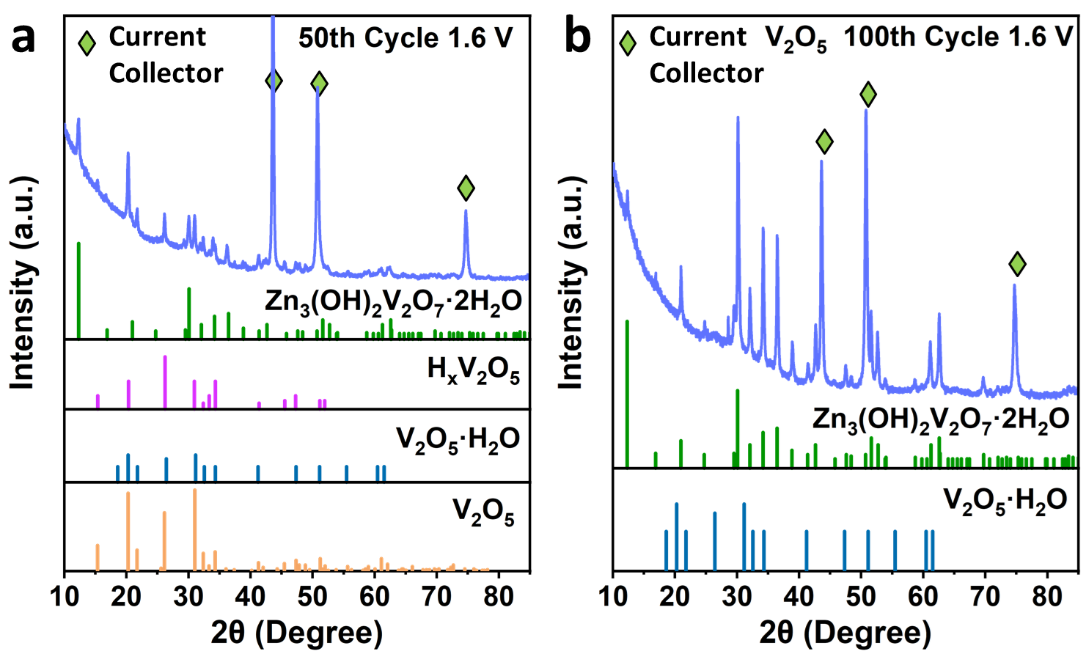


**Figure S10.** XRD patterns of V_2_O_5_ cathodes in real coin cells at charging state at 50^th^ cycle (a) and 100^th^ cycle (b).

Standard card: PDF#50-0570 corresponds to Zn_3_(OH)_2_V_2_O_7_·2H_2_O; PDF#45-0429 corresponds to H_x_V_2_O_5_; PDF#21-1432 corresponds to V_2_O_5_·H_2_O; PDF#41-1426 corresponds to V_2_O_5_.


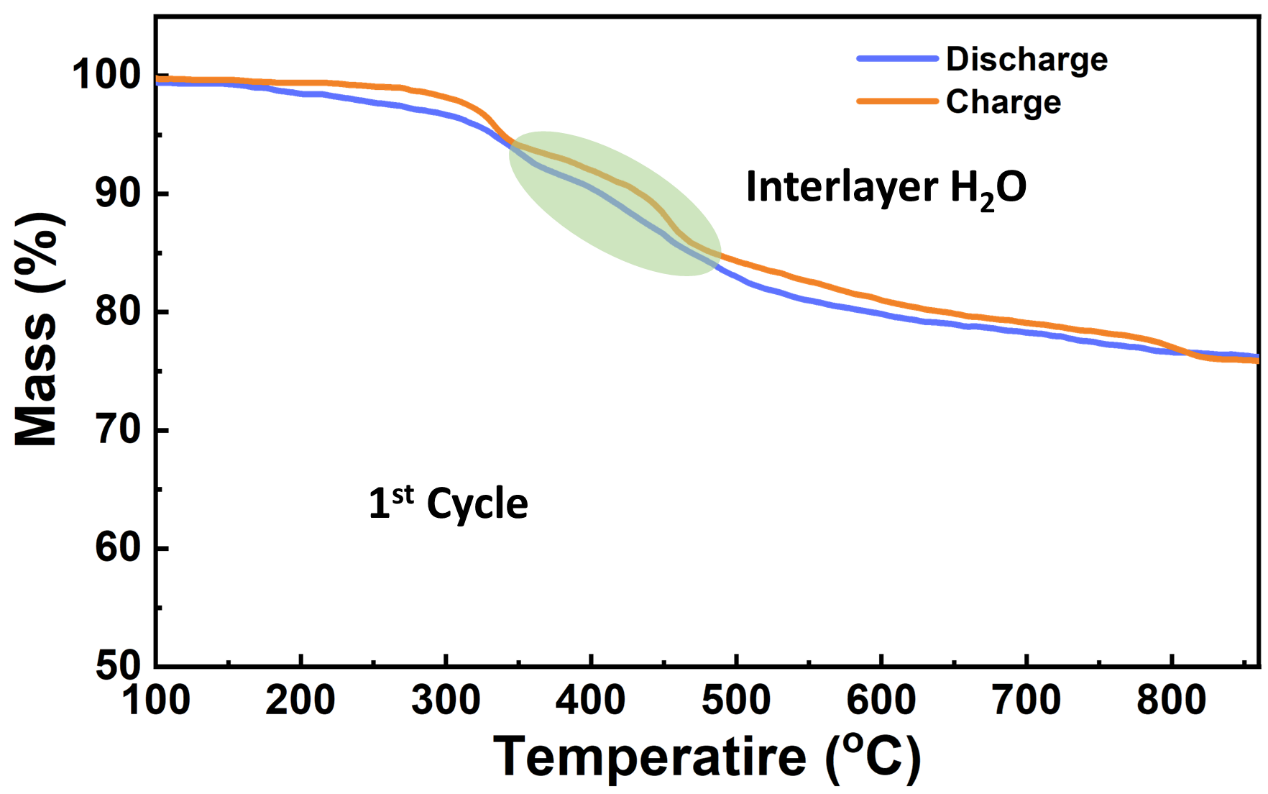


**Figure S11.** Thermogravimetry (TG) curves of the electrode at the first cycle.

Before the test data was recorded, the free water was removed at 100 ^o^C for 40 minutes, and the weight loss in the temperature range of 350-500 ^o^C was interlayer water. ^[4]^


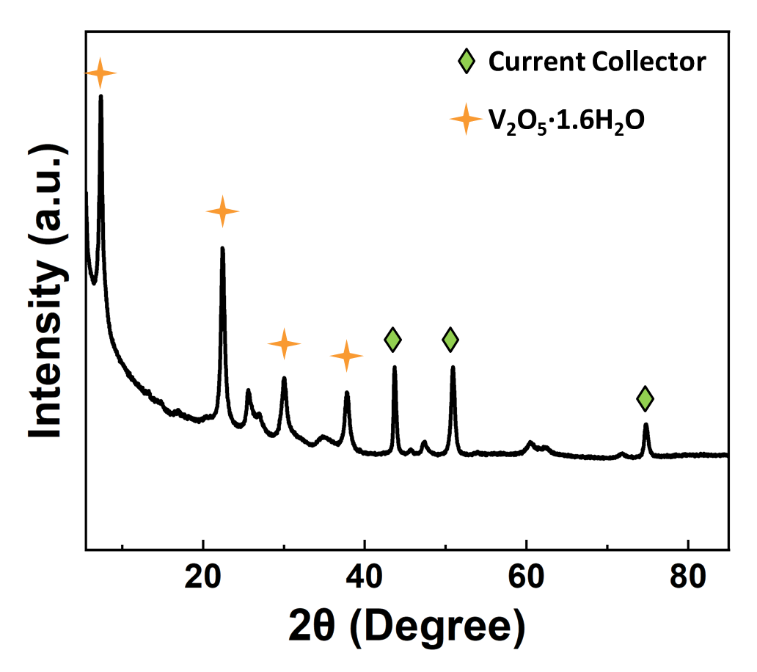


**Figure S12.** XRD pattern of V_2_O_5_·1.6H_2_O electrode.


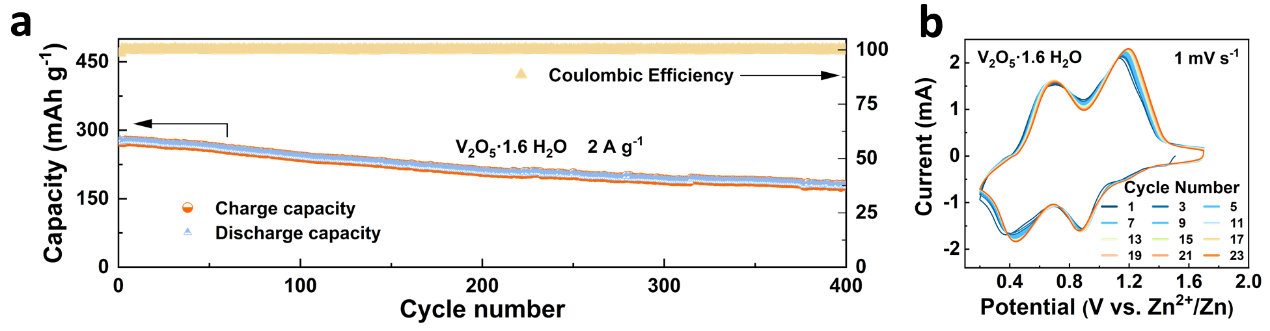


**Figure S13.** Zn/V_2_O_5_·1.6H_2_O real coin cells’ long cycle performance at 2 A g^−1^ (a) and cyclic voltammetry curves at 1 mV s^−1^ (b).

There is no capacity increase process in the Zn/V_2_O_5_·1.6H_2_O coin cell. Similarly, the CV curves maintain nearly identical peak positions and intensities with increasing cycle numbers, which is similar to the long cycle CV curves of V_2_O_5_. This indicates that the capacity increase is due to the gradual transition of V_2_O_5_ to V_2_O_5_·nH_2_O.


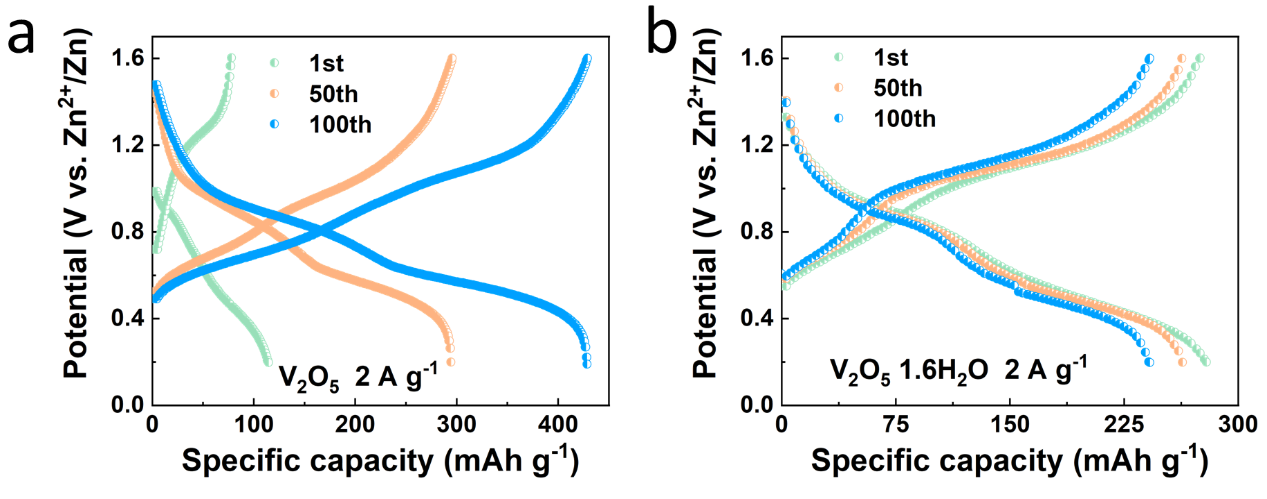


**Figure S14.** Comparisons of charge/discharge curves at different cycle stages: (a) Zn/V_2_O_5_ real coin cell. (b) Zn/V_2_O_5_·1.6H_2_O real coin cell.

At the beginning of the cycle, the polarization of the charge/discharge curves of Zn/V_2_O_5_ system are greater than Zn/V_2_O_5_·1.6H_2_O system, and gradually decreases with the progress of the cycle. The charge/discharge curves of Zn/V_2_O_5_ system gradually close to that of Zn/V_2_O_5_·1.6H_2_O system.


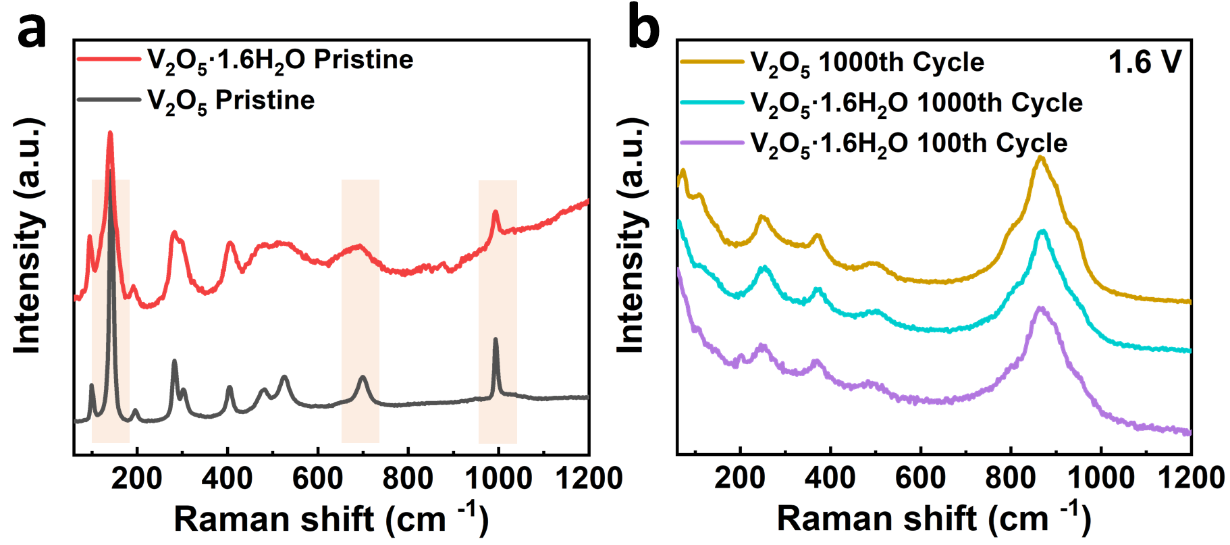


**Figure S15.** The Raman peaks of pristine V_2_O_5_·1.6H_2_O are similar to those of V_2_O_5_. (b) The Raman spectra of V_2_O_5_ and V_2_O_5_·1.6H_2_O after 100 cycles at charge to 1.6 V.

After 100 cycles, both Zn/V_2_O_5_ and Zn/V_2_O_5_·1.6H_2_O systems exhibit identical peak shapes, indicating that these two materials ultimately transformed into the same phase after prolonged cycling. The peaks at 251, 370, 495, and 870 cm^−1^ can be attributed to the Zn−OH vibration of Zn_3_(OH)_2_V_2_O_7_·2H_2_O.^[2]^ (Figure S15b).


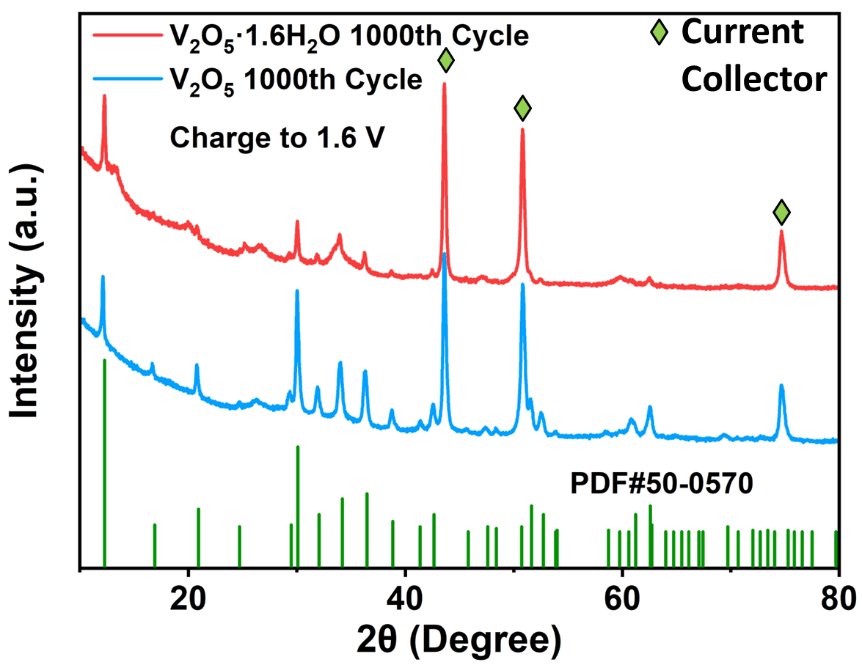


**Figure S16.** After a long cycle, the XRD patterns of the V_2_O_5_ cathode and the V_2_O_5_·1.6H_2_O cathode are in good correspondence with the PDF standard card (PDF#50-0570) of Zn_3_(OH)_2_V_2_O_7_·2H_2_O.


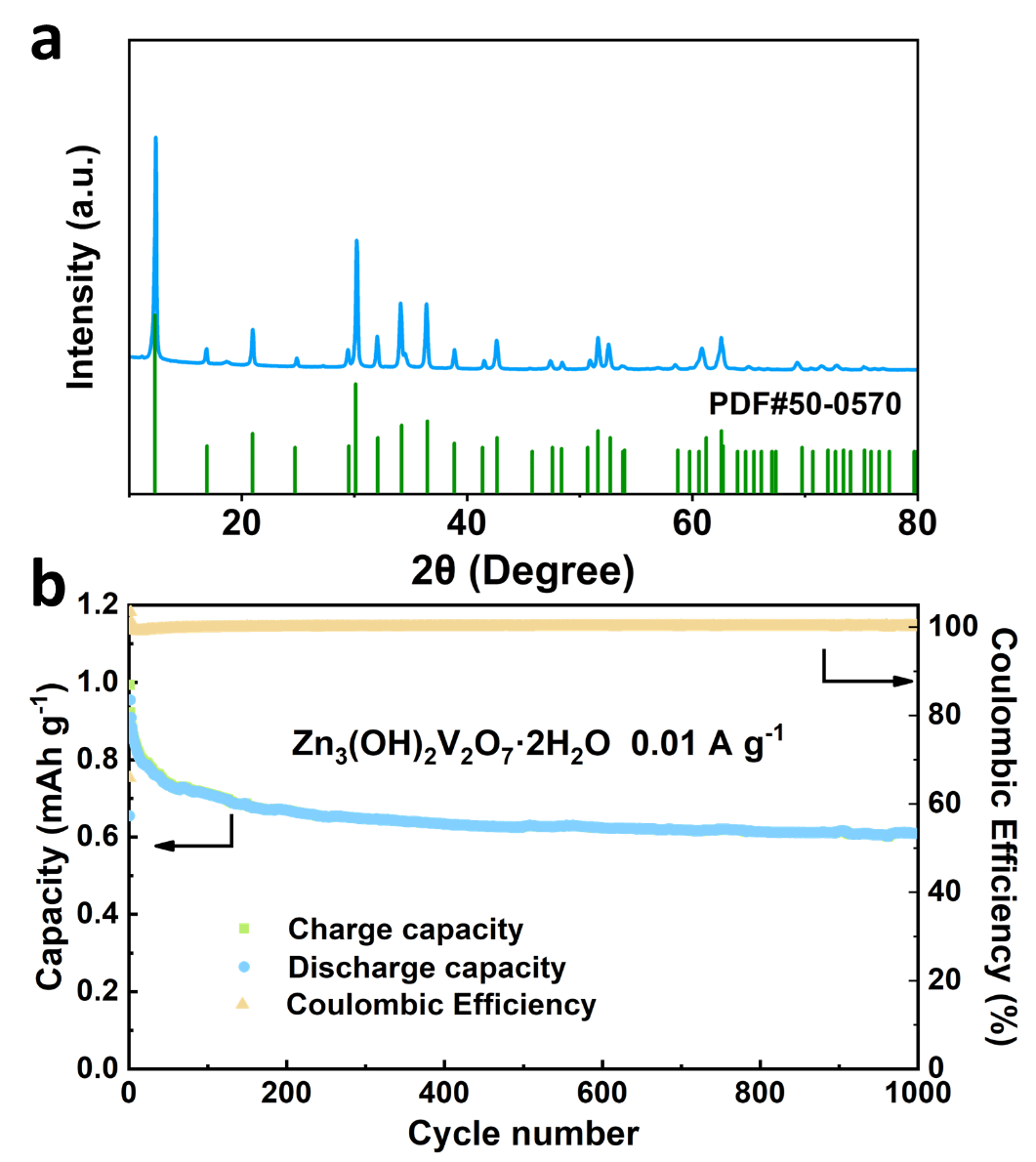


**Figure S17.** (a) The XRD pattern of Zn_3_(OH)_2_V_2_O_7_·2H_2_O prepared by a hydrothermal method is in good correspondence with its PDF standard card (PDF#50-0570). (b) The performance of Zn/Zn_3_(OH)_2_V_2_O_7_·2H_2_O battery at 0.01 A g^-1^.

We synthesized pure Zn_3_(OH)_2_V_2_O_7_·2H_2_O by hydrothermal method (Figure S17a) as the cathode to assemble the battery with a zinc anode. The results show that the battery capacity is negligible (less than 1 mA h g^-1^ at 0.01 A g^-1^, Figure S17b), which further supports the notion that capacity decay after prolonged cycling is attributed to Zn_3_(OH)_2_V_2_O_7_·2H_2_O formation.

**
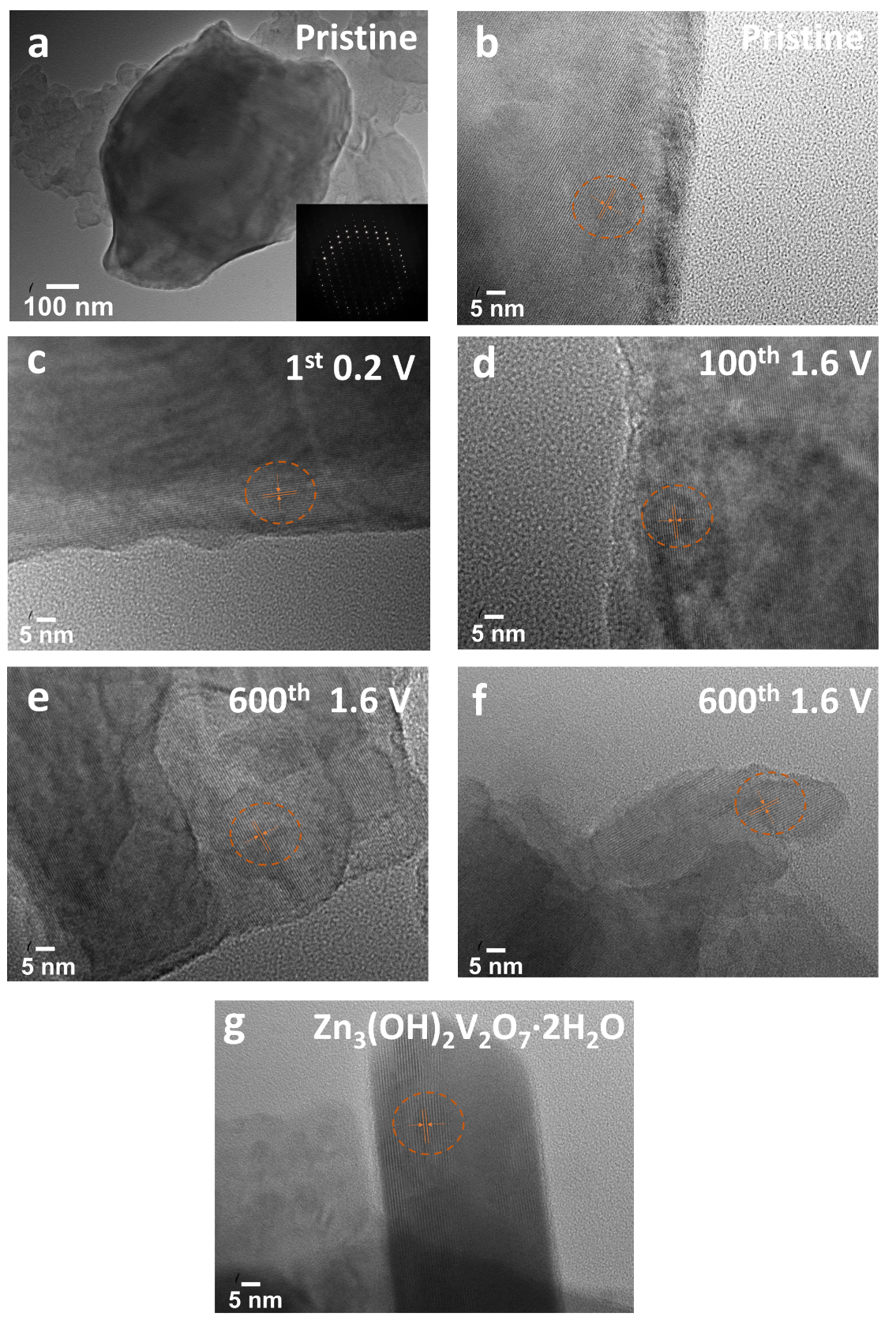
**

**Figure S18.** (a) TEM images of pristine V_2_O_5_ and its corresponding SAED pattern. (b-f) HRTEM images of pristine V_2_O_5_ and cycled at different states. (g) HRTEM image of prepared Zn_3_(OH)_2_V_2_O_7_·2H_2_O.

The yellow circles in (b-g) correspond to the areal selected for HRTEM imaging to detect their lattice spacings, as shown in Figure 1f-j.


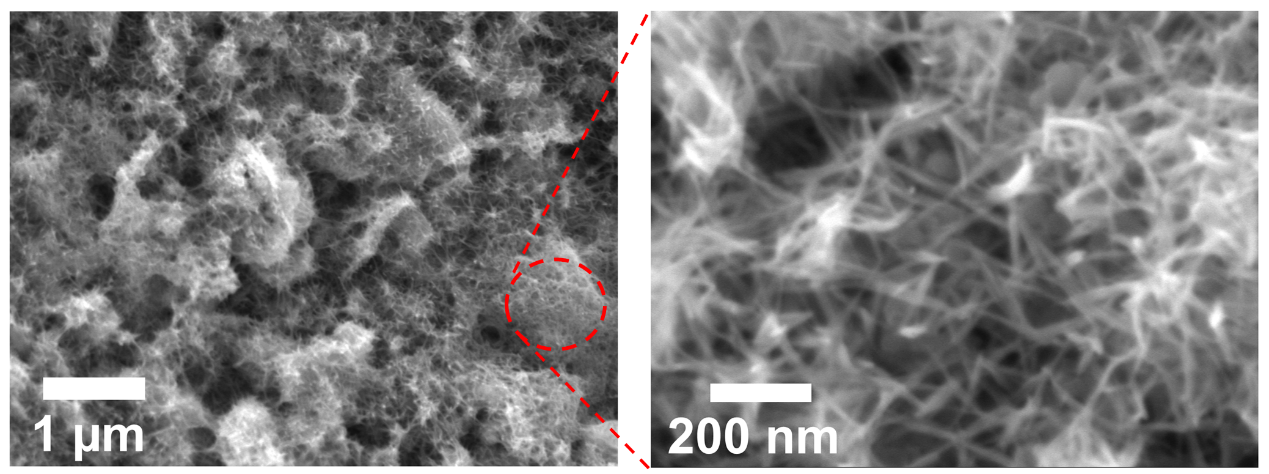


**Figure S19.** FESEM images of the cathode surface at the 100^th^ cycle. On the surface of large particles, many sheet structures are generated.


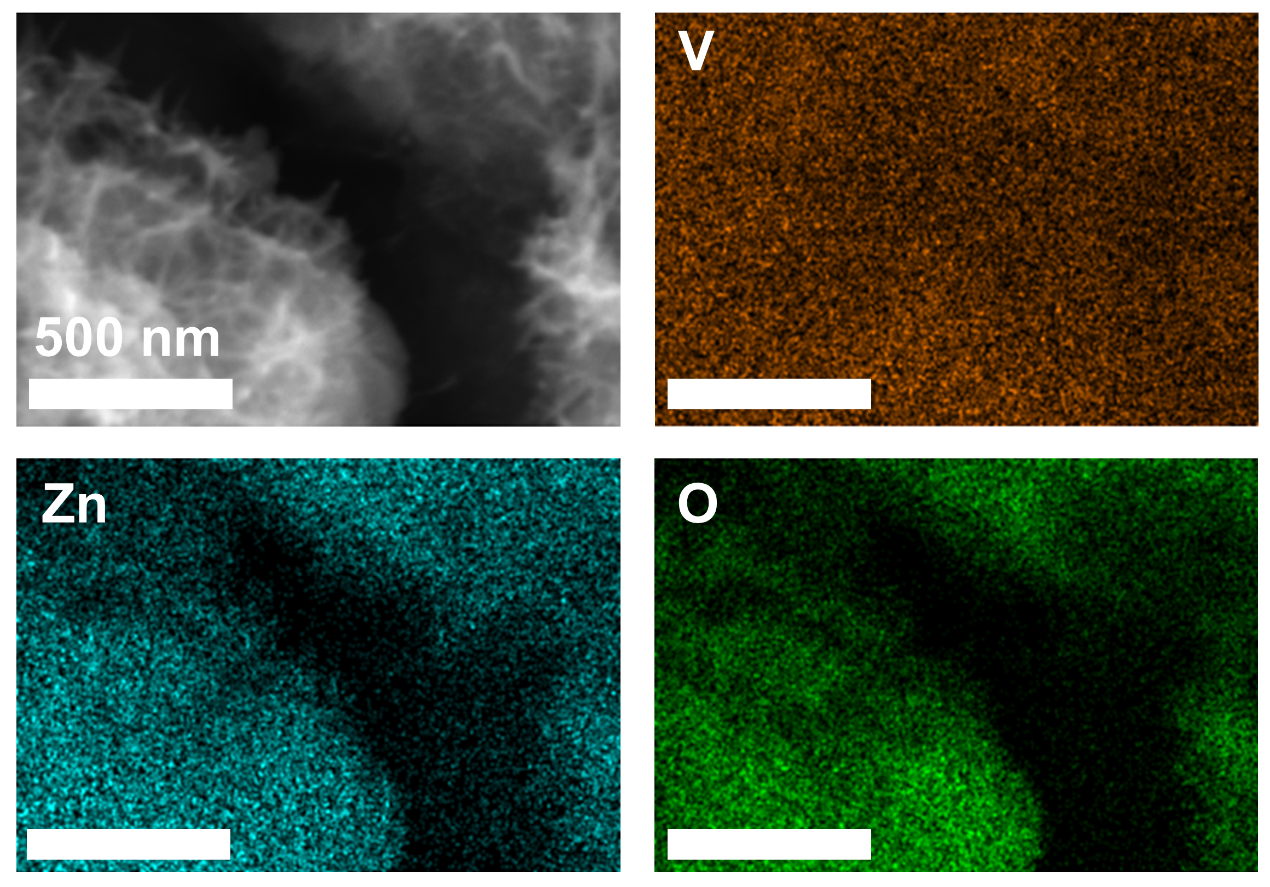


**Figure S20.** EDS elemental mapping images of the cathode after 100 cycles at full charge state (1.6 V).


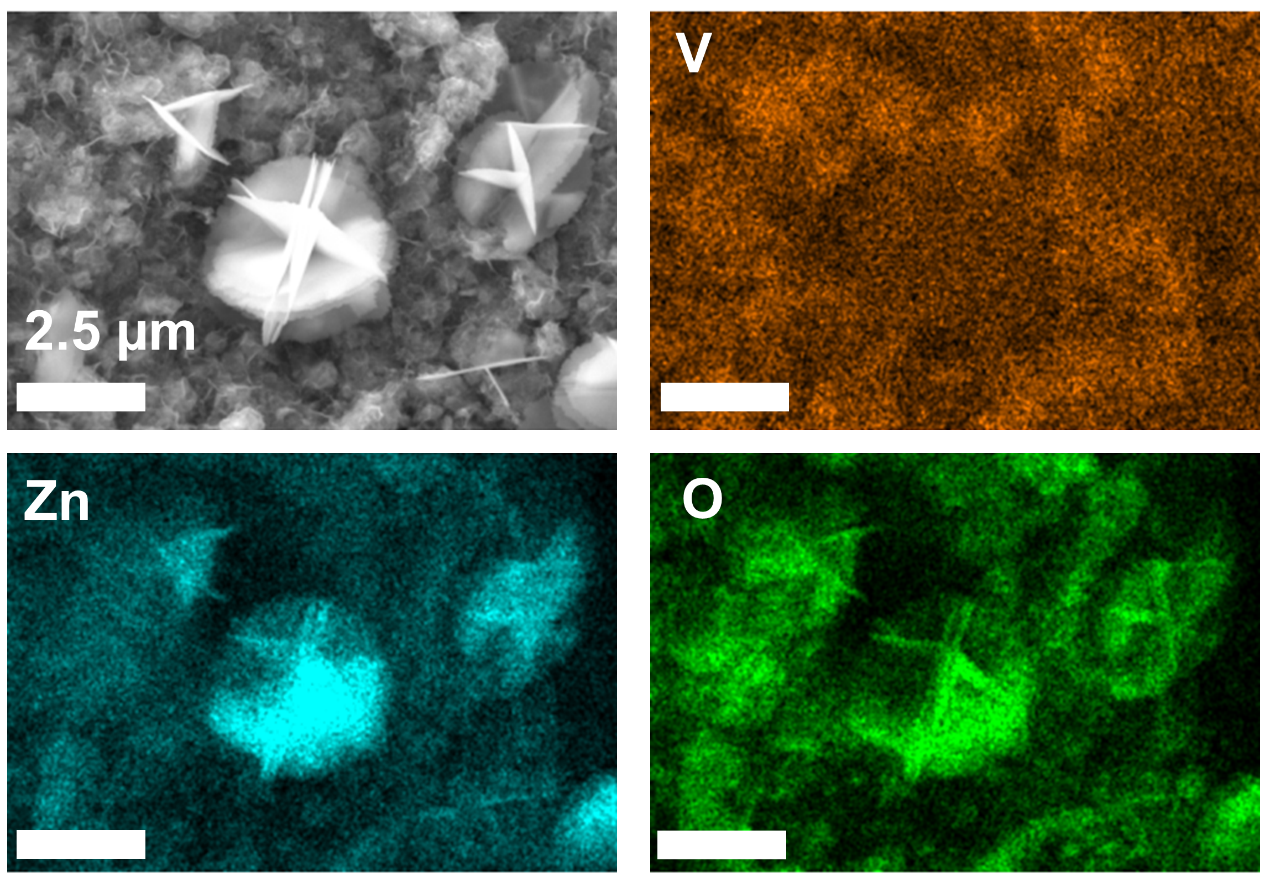


**Figure S21.** EDS mapping images of the cathode after 600 cycles at full charge state (1.6 V).


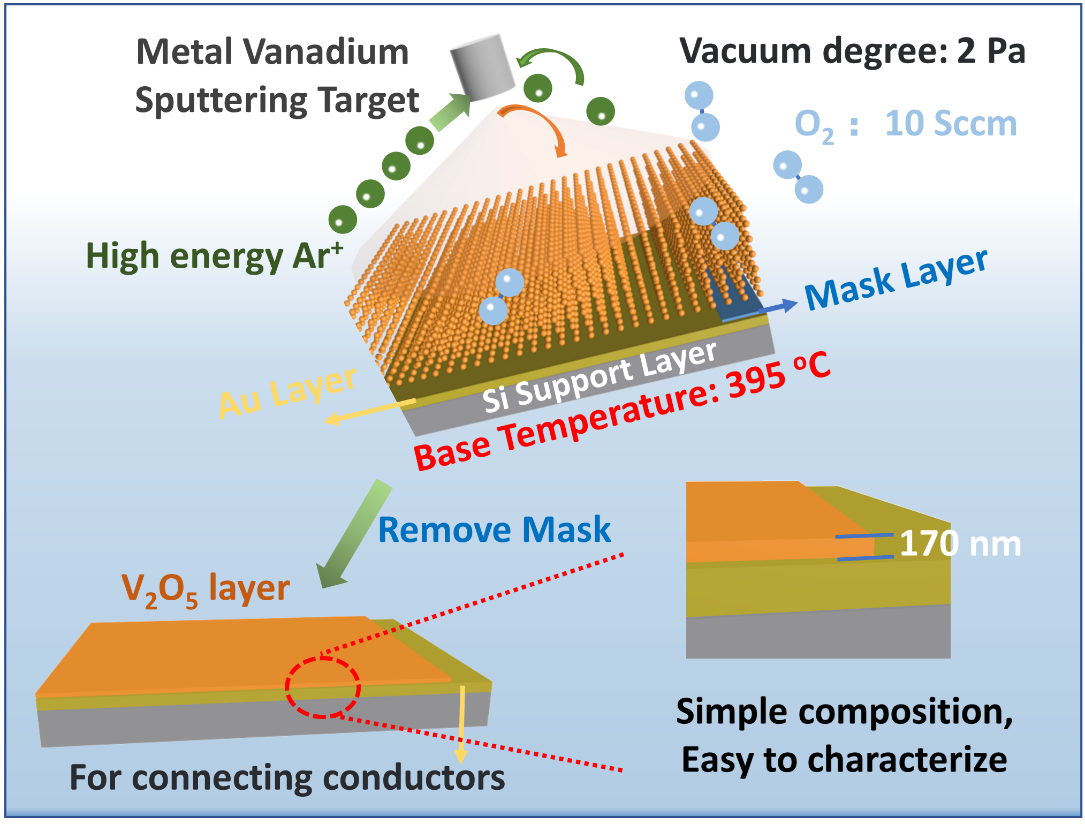


**Figure S22.** Schematic diagram of film electrode preparation processes with a single component and regular structure by magnetron sputtering.

Thanks to the simple component and regular layered structure of the film electrode, there are neither other complex components nor the interferences that absorbed residual electrolytes between stacked active material particles in a real coin electrode. As long as the surface of the film electrode is properly cleaned, precise characterization of the measured sample can be achieved. This process, known as "interference-free characterization", enables obtaining authentic information without any contaminations.


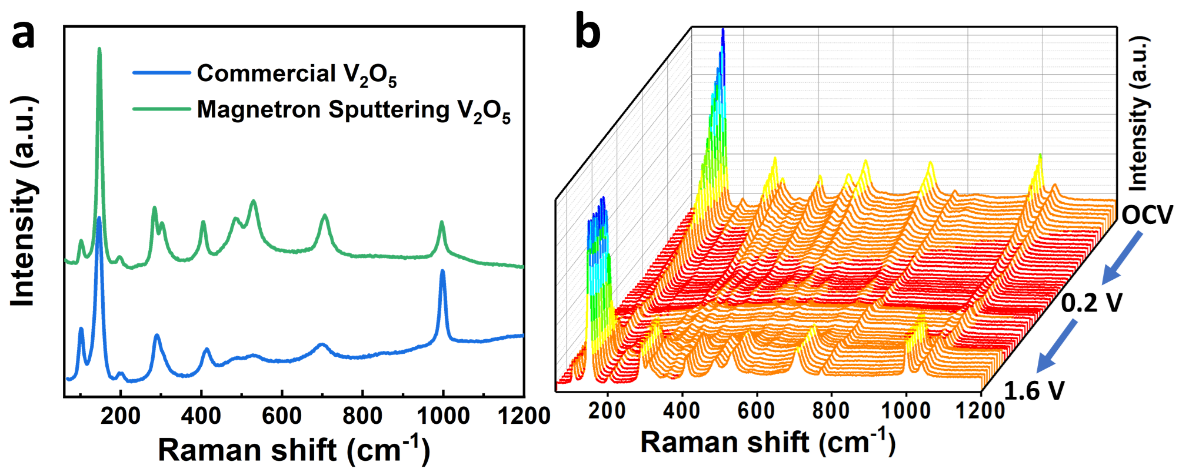


**Figure S23.** (a) Comparisons of Raman spectra of commercial V_2_O_5_ and prepared V_2_O_5_ by magnetron sputtering. (b) 3D pattern chart of operando Raman spectra of the first discharge/charge cycle of Zn/V_2_O_5_ model battery.

**
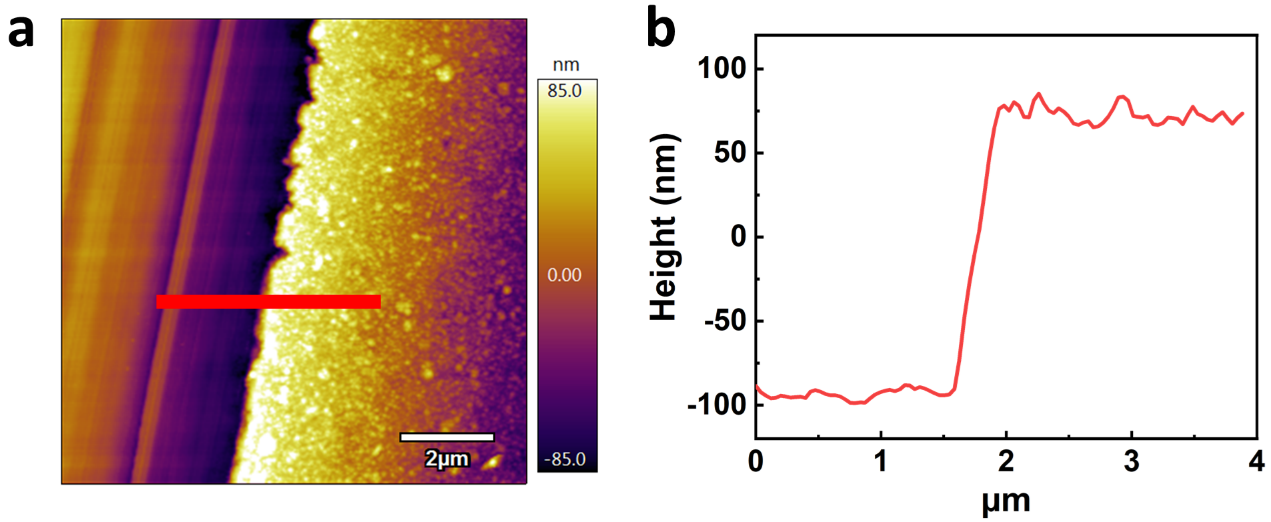
**

**Figure S24.** The thickness of the V_2_O_5_ layer of film electrode measured by AFM is about 170 nm.


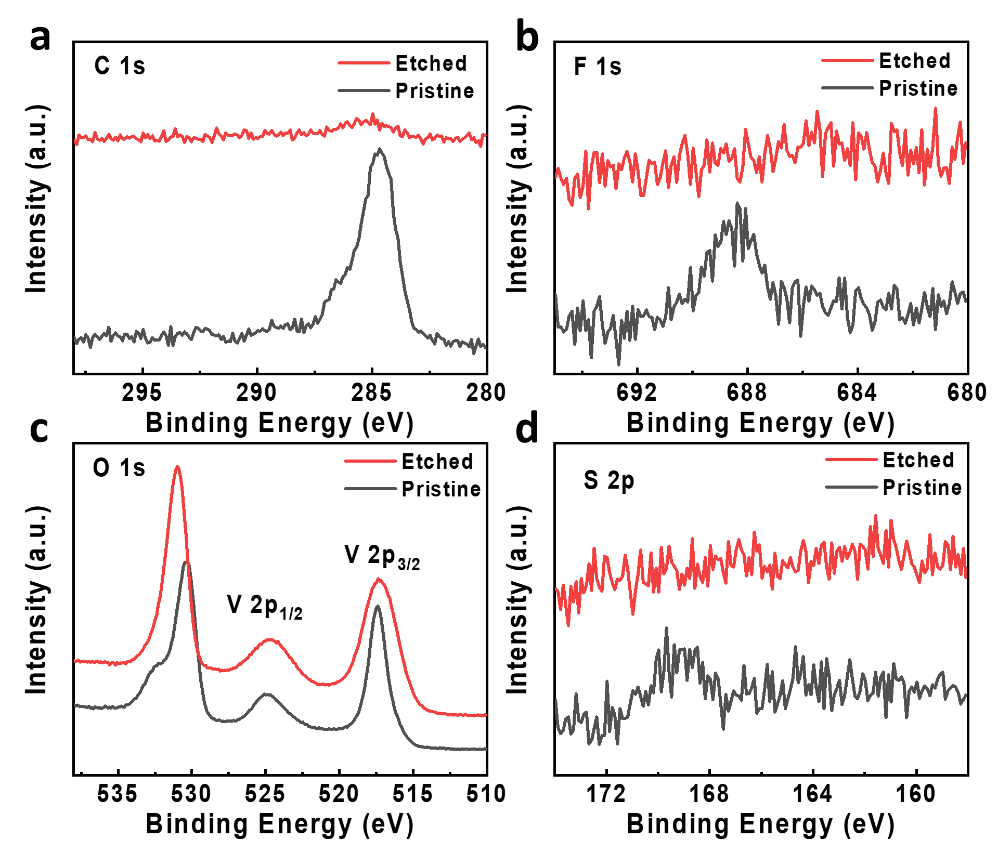


**Figure S25.** Under optimized etching parameters (GCIB, 5 kV, 2.5 min), the comparisons of XPS spectra of each element before and after etching: C 1s (a), F 1s (b), O 1s (c), S 2p (d).

**
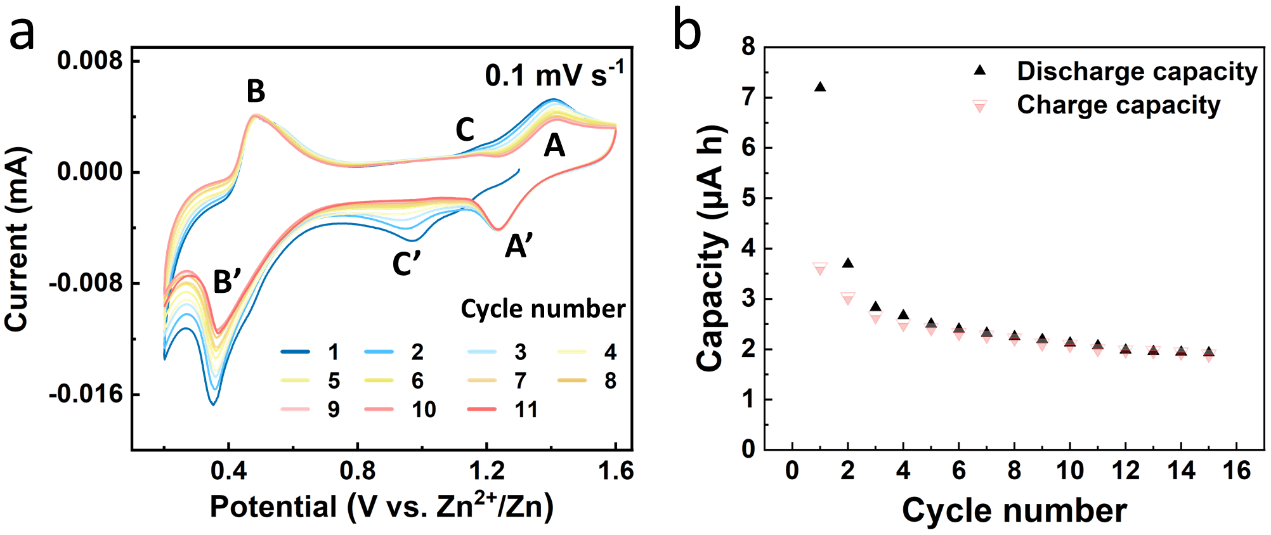
**

**Figure S26.** V_2_O_5_ film cathode’s long cycle CV curves at 0.1 mV s^−1^ (a) and capacity evolution (b).

The CV curves of film cathode show a similar evolution trend to that of the real powder electrode. There are changes from initial 3 pairs of peaks evolving to 2 pairs of peaks, corresponding to the transformation of V_2_O_5_ to V_2_O_5_·nH_2_O. However, since the thickness of the thin film electrode (170 nm in Figure S24) is much smaller than the particle size of the actual electrode (1-3 µm in Figure 1k), the activation process is quickly completed. The capacity loss caused by V dissolution accounts for a larger proportion, so the peak current in CV curves is gradually reduced, corresponding to the gradual reduction of the capacity. At the same time, considering that the capacity in the subsequent activation process is constantly changing due to the accumulation of H_2_O molecules in the layers, the studies of the intercalation chemistry are based on the first cycle of electrodes.


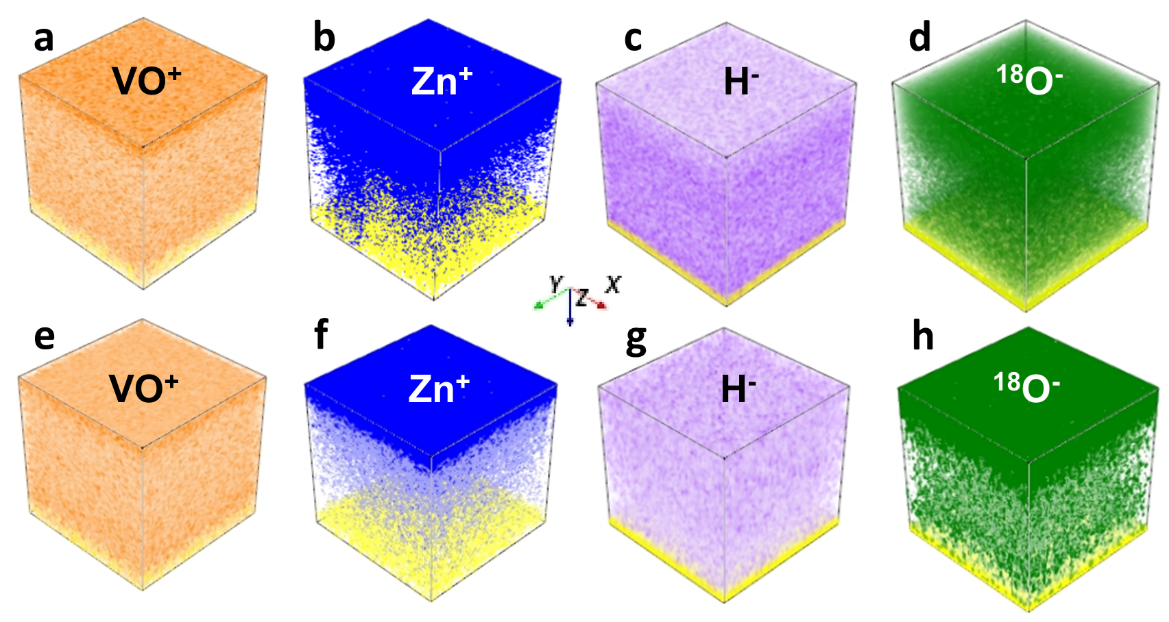


**Figure S27.** TOF-SIMS results of film electrode: at the 10^th^ cycle discharging to 0.2 V (a-d) and charging to 1.6 V (e-h).

**Table S1**

|  | O_L_ (%) | -OH (%) | H_2_O (%) |
| --- | --- | --- | --- |
| Discharge to 0.6 V | 49.20 | 34.50 | 16.30 |
| Discharge to 0.2 V | 36.01 | 33.29 | 30.70 |
| Charge to 1.0 V | 55.67 | 24.07 | 20.26 |
| Charge to 1.6 V | 63.22 | 19.47 | 17.31 |

The proportions of O 1s species in Figure 4c-f are as follows: O_L_: -OH: H_2_O = 49.20: 34.50: 16.30, 36.01: 33.29: 30.70, 55.67: 24.07: 20.26, 63.22: 19.47: 17.31.


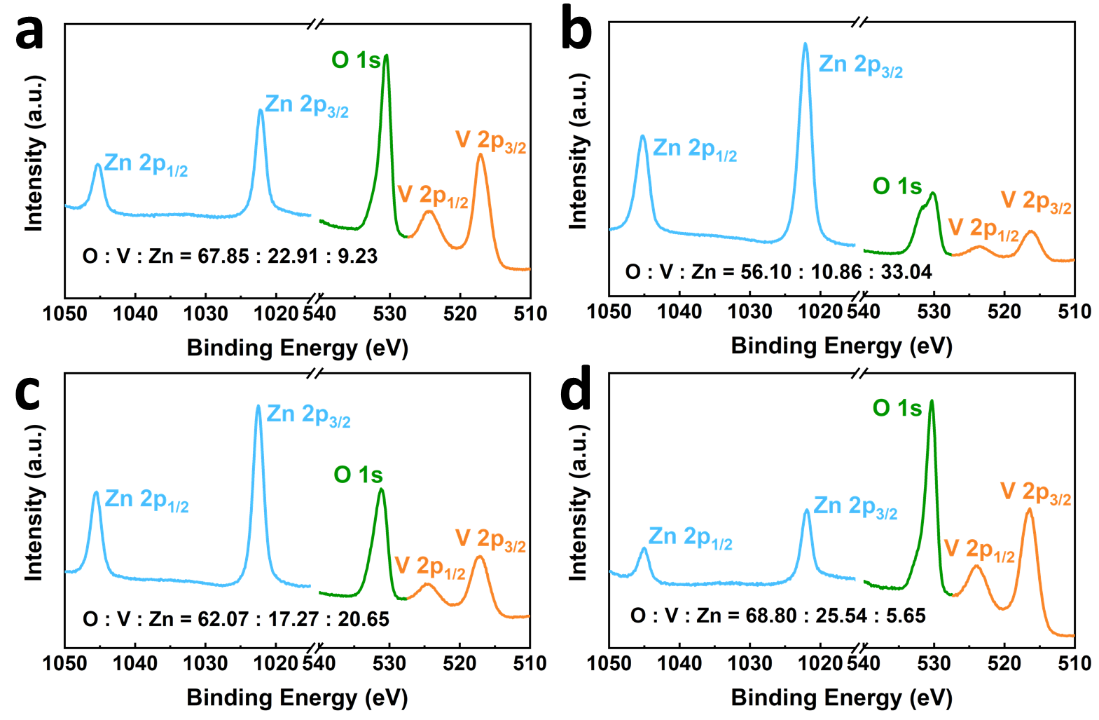


**Figure S28.** Comparisons of O, V, and Zn elements in the model electrodes: discharged to 0.6 V (a), 0.2 V (b), charged to 1.0 V (c), 1.6 V (d).

**Table S2**

|  | O (%) | V (%) | Zn (%) |
| --- | --- | --- | --- |
| Discharge to 0.6 V | 67.85 | 22.91 | 9.23 |
| Discharge to 0.2 V | 56.10 | 10.86 | 33.04 |
| Charge to 1.0 V | 62.07 | 17.27 | 20.65 |
| Charge to 1.6 V | 68.80 | 25.54 | 5.65 |

The proportions of zinc species in Figure S28 a-d are as follows: O: V: Zn = 67.85: 22.91: 9.23, 56.10: 10.86: 33.04, 62.07: 17.27: 20.65, 68.80: 25.54: 5.65.


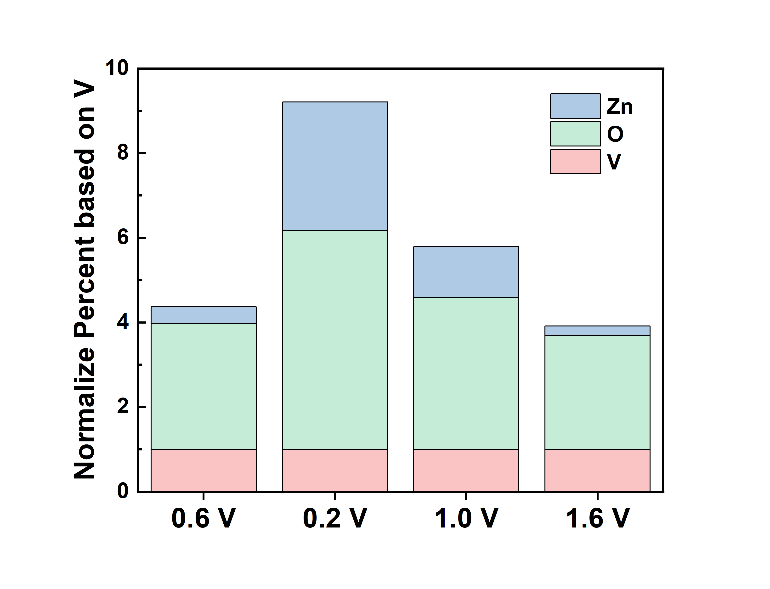


**Figure S29.** Histogram comparison of O, V, and Zn concentration ratio at different voltages.


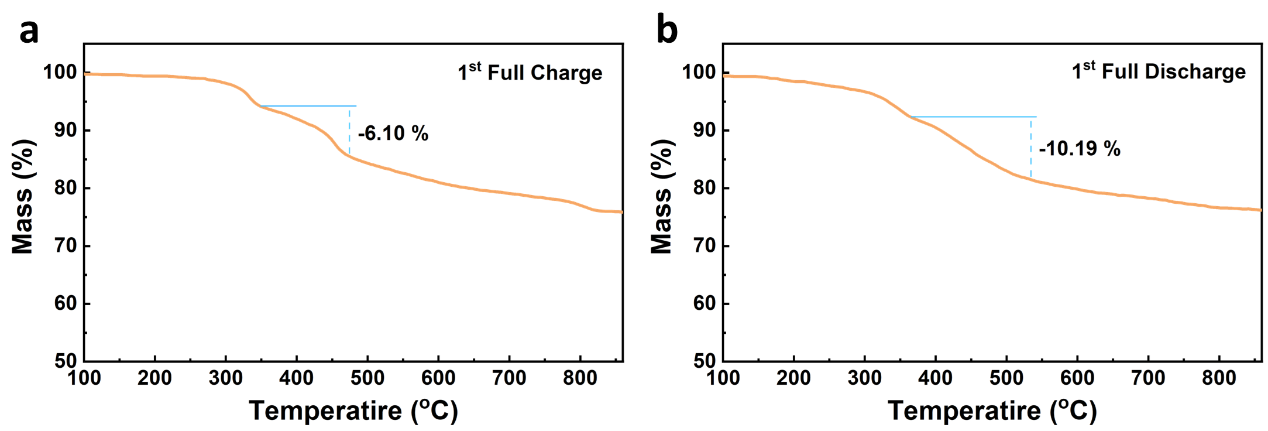


**Figure S30.** Thermogravimetry (TG) curves of the electrode at the full charge to 1.6 V (a) and full discharge to 0.2 V (b) at 1^st^ cycle.

Before the test data was recorded, the free water was removed at 100 ^o^C for 40 minutes, and the weight loss in the temperature range of 350-500 ^o^C was interlayer water. ^[4]^


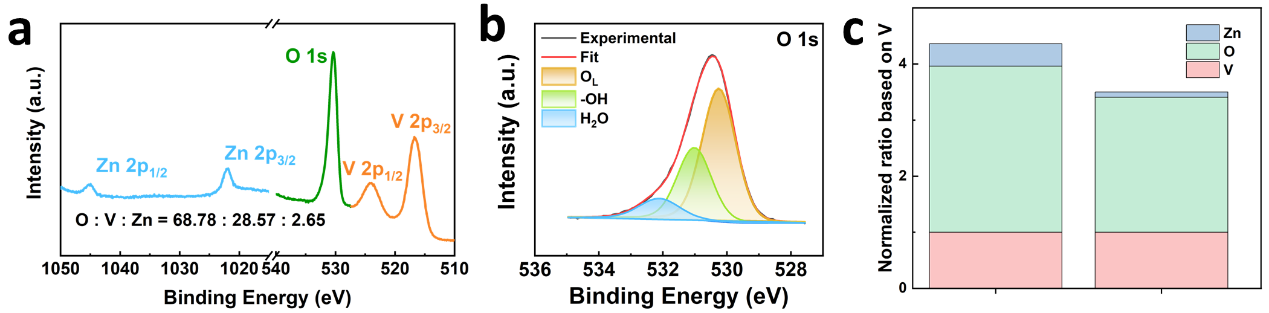


**Figure S31.** (a, b) Comparisons of XPS spectra of O, V, and Zn signals and XPS O 1s spectra in the film electrode soaked for the same duration as the discharge to 0.6 V. (c) The atomic concentration ratios of O, V, and Zn species normalized based on their respective V element.

For the sample discharged to 0.6 V, the O: V: Zn ratio was 67.85: 22.91: 9.23, as shown in Figure 5a. While for the sample that was only immersed in the electrolyte, the O: V: Zn ratio was 68.78: 28.57: 2.65 (Figure S31a). The results indicate that Zn^2+^ can be inserted into the layers only by immersion, while the presence of an electric field enhances the intercalation of Zn^2+^, resulting in 3.48 times the amount of intercalation compared to when no electric field is applied. The O 1s spectra reveal that the proportions of O_L_: -OH: H_2_O are 49.20: 34.50: 16.30 and 55.96: 33.35: 10.69, respectively with and without an electric field. These results suggest that the presence of an electric field promotes the intercalation of ions and H_2_O molecules, thereby facilitating the phase transition of V_2_O_5_ during the battery cycling. If the soaking time is extended to 36 h (Figure 5b, d), the Zn signals are greatly enhanced. The ratio of O: V: Zn is 60.60: 11.06: 28.34, and the content of Zn is about 6.36 times that of discharge to 0.6 V. The results show that prolonged soaking time would promote the interlayer intercalation of Zn^2+^.


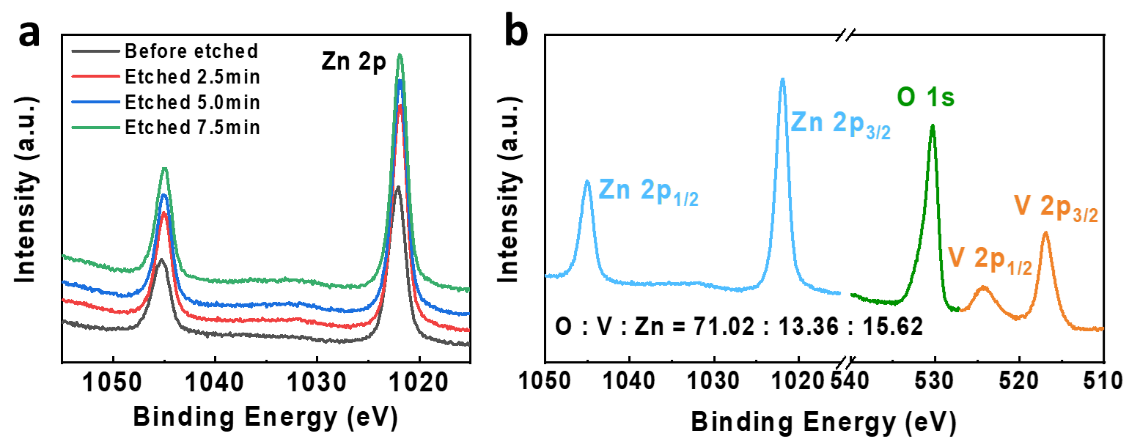


**Figure S32.** (a) Depth-dependent XPS spectra of Zn 2p of film electrode charged to 1.6 V at 50^th^ cycle. (b) Ration of O: V: Zn after etching 2.5 min.

The depth-dependent XPS characterization was performed on the film electrode after it was charged to 1.6 V during the 50^th^ cycle. The results showed that the Zn 2p signal remains robust even after etching for 7.5 minutes (Figure S32a). The O: V: Zn ratio was 71.02: 13.36: 15.62, as shown in Figure S32b, the Zn^2+^ content is 5.29 times that of the first round of charging, which is consistent with the incomplete deintercalation of Zn^2+^ in the charging state observed in intercalation chemistry studies. This indicates that the formation of Zn_3_(OH)_2_V_2_O_7_·2H_2_O is attributed to the gradual accumulation of irreversibly extracted Zn^2+^ in the interlayer during battery cycling.


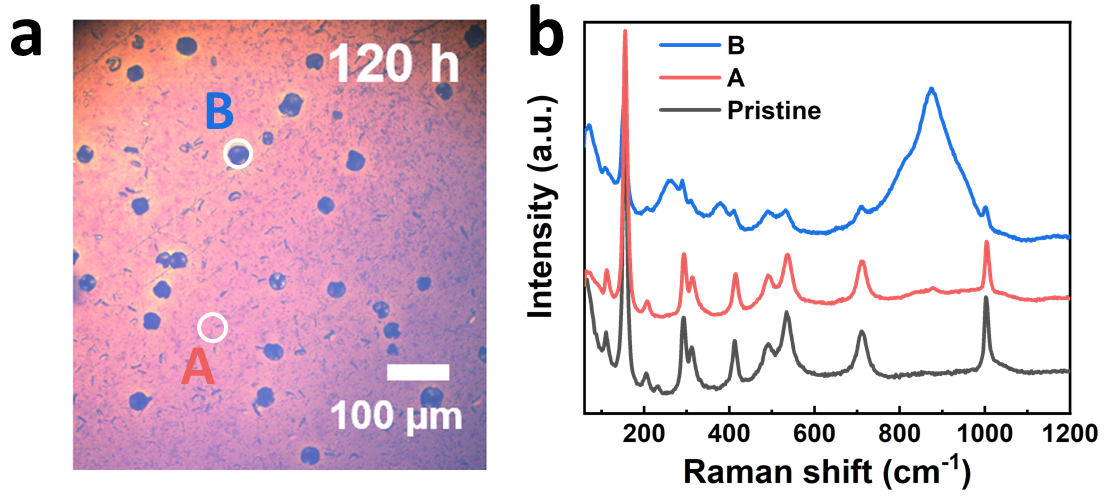


**Figure S33.** (a) The Optical microscope photograph of the pristine model electrode was soaked in 3M Zn(OTf)_2_ aqueous solution for 120 hours. (b) Raman spectra comparisons in photograph a. The blue line observed Raman shifts at 370, 495, and 870 cm^−1^ can be attributed to the Zn−OH vibration of Zn_3_(OH)_2_V_2_O_7_·2H_2_O.^[2]^

Slight dissolution of V_2_O_5_ during soaking and subsequent chemical reactions with Zn^2+^, might also contribute to the transformation. The reactions are summarized via the following reactions.^[3]^

Dissolution: V_2_O_5_ + 3 H_2_O → 2 VO_2_(OH)_2_^−^ + 2 H^+^ (1)

Precipitation: 2 VO_2_(OH)_2_^−^ + 3 Zn^2+^ + 3 H_2_O → Zn_3_(OH)_2_V_2_O_7_·2H_2_O + 4 H^+^ (2)

0.2 g V_2_O_5_ was added in 20 mL of different solutions. The above mixtures were stirred for 48 hours. In an acidic environment, V_2_O_5_ is slightly soluble. Therefore, the above mixtures were filtered out of the undissolved V_2_O_5_ to obtain the filtrate. The pH of the filtrate before and after dissolution is measured. We took 500 µL of the above filtrates and diluted them into 20 mL water. The content of V measured by ICP is listed in Table S3-5.

**Table S3**

|  | **H_2_O** | **3 mol kg^-1^ Zn(OTf)_2_** | **3 mol kg^-1^ Zn(OTf)_2_+HOTf** | **3 mol kg^-1^ Zn(OTf)_2_+HOTf** |
| --- | --- | --- | --- | --- |
| Initial pH | 6.60 | 3.94 | 1.85 | 0.41 |
| Stir 48 h pH | 2.60 | 2.04 | 1.30 | 0.72 |
| **V content (mg L^-1^)** | **12.91** | **1.59** | **0.86** | **0.70** |

According to Table S3-1, once Zn(OTf)_2_ is dissolved in water (whether the pH is adjusted to 1.85 or 0.41), its capacity to dissolve vanadium decreases. The addition of HOTf further inhibits vanadium dissolution. When the initial pH is greater than 1.85, the pH decrease can be attributed to **reaction (S1)**: V_2_O_5_ + 3 H_2_O → 2 VO_2_(OH)_2_^−^ + 2 H^+^, where VO_2_(OH)_2_^−^ becomes the stable V-species, releasing protons. This VO_2_(OH)_2_^−^ species can then react with Zn^2+^ to form Zn_3_(OH)_2_V_2_O_7_·2 H_2_O, as shown in **reaction (S2)**: 2 VO_2_(OH)_2_^−^ + 3 Zn^2+^ + 3 H_2_O → Zn_3_(OH)_2_V_2_O_7_·2 H_2_O + 4 H^+^. When the initial pH is less than 0.41, the pH increases after dissolution, the stable V-species in the solution is VO_2_^+^ accompanied by consuming protons: V_2_O_5_ + 2 H^+^ → 2 VO_2_^+^ + 2 H_2_O **reaction (S3)**.

**Table S4**

|  | **H_2_O** | **3 mol kg^-1^ ZnSO_4_** | **3 mol kg^-1^ ZnSO_4_+** **H_2_SO_4_** | **3 mol kg^-1^ ZnSO_4_+HOTf** |
| --- | --- | --- | --- | --- |
| Initial pH | 6.60 | 4.33 | 1.40 | 0.10 |
| Stir 48 h pH | 2.60 | 2.11 | 1.52 | 0.57 |
| **V content (mg L^-1^)** | **12.91** | **5.69** | **14.26** | **91.25** |

According to Table S4, when ZnSO_4_ is dissolved in water, its capacity to dissolve vanadium also decreases. However, the solubility of 5.69 mg L^-1^ in ZnSO_4_ system is higher than that of 1.59 mg L^-1^ in Zn(OTf)_2_ system (Table S3), indicating that the anion type affects the solubility of V_2_O_5_. Unlike in Zn(OTf)_2_ system, whether H_2_SO_4_ or HOTf is added, it will promote the dissolution of vanadium, the pH increases after dissolution, and the stable V-species in the solution is VO_2_^+^ accompanied by consuming protons: V_2_O_5_ + 2 H^+^ → 2 VO_2_^+^ + 2 H_2_O **reaction (S3)**.

**Table S5**

|  | **H_2_O** | **H_2_O +**  **H_2_SO_4_** | **H_2_O +**  **H_2_SO_4_** | **H_2_O +** **HOTf** | **H_2_O +HOTf** |
| --- | --- | --- | --- | --- | --- |
| Initial pH | 6.60 | 0.67 | 0.96 | 0.81 | 1.17 |
| Stir 48 h pH | 2.60 | 0.35 | 1.20 | 0.41 | 1.41 |
| **V content (mg L^-1^)** | **12.91** | **218.61** | **41.30** | **80.80** | **22.49** |

According to Table S5, when only H_2_SO_4_ or HOTf is used to adjust the pH of the solution without introducing other cations, the dissolution of V_2_O_5_ is facilitated. According to the pH changes before and after dissolution, it is evident that both mechanisms involving proton consumption and proton generation exist.

Based on the above results, it is shown that the solubility of V_2_O_5_ is greatly affected by the change in pH, which is not completely consistent with the phase diagram reported in the literature^[5]^ because of the influence of anions and cations.


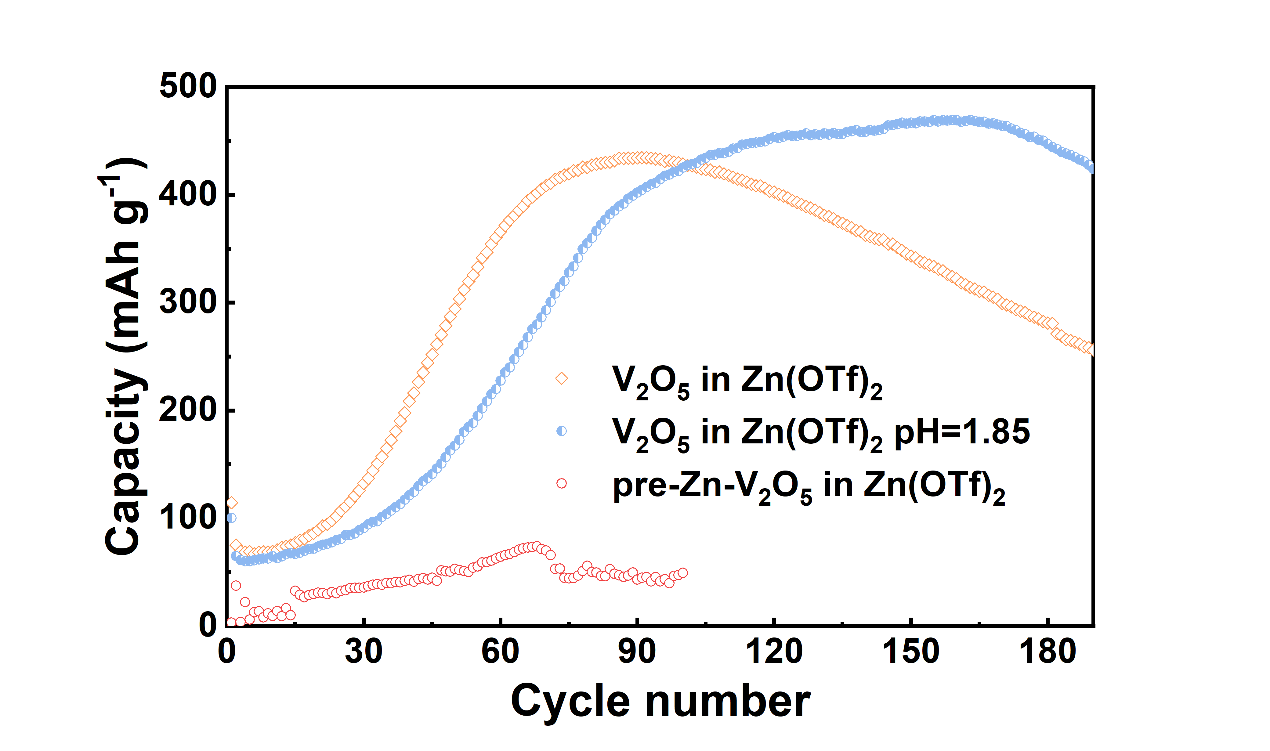


**Figure S34.** Capacity comparisons of battery performance in different systems.

The blue line in Figure S34 represents the battery performance of the V_2_O_5_ cathode when the pH of the 3M Zn(OTf) electrolyte is adjusted to 1.85. Compared with the original electrolyte (pH=3.94, orange line), the peak capacity of the battery is higher and the peak duration is longer in case of pH to 1.85. And the activation process is longer can be explained as follows: the residual H_2_O molecules in the interlayer of cathode during activation are mainly derived from the co-intercalated H_2_O molecules with Zn^2+^ ions. After adding HOTf acid to the electrolyte, the preferential insertion of protons reduces the proportion of zinc ion intercalation, thus reducing the amount of co-intercalation water molecules, and thus prolonging the activation process. The cathode material used in the red line system is called “pre-Zn-V_2_O_5_”, which is prepared by soaking V_2_O_5_ powder in 3M Zn(OTf) electrolyte and stirring it for 24 hours. Due to the immersion process, Zn^2+^ were pre-embedded and accumulated in the V_2_O_5_ layers, resulting in a very low capacity.

**References**

[1] a) K Y. Zhang, X. Jing, Y. Cheng, T. Hu, C. Meng, *Inorg. Chem. Front.* **2018,** *5*, 2798-2810. b) Y. Song, T. Liu, B. Yao, T. Kou, D. Feng, X. Liu, Y. Li, *Small,* **2017,** *13,* 1700067*.*

[2] a) W. Dong, M. Du, F. Zhang, X. Zhang, Z. Miao, H. Li, Y. Sang, J. Wang, H. Liu, S. Wang, *ACS Appl. Mater. Interfaces* **2021,** *13*, 5034-5043; b) HD. Lutz, C. Jung, R. Mörtel, H. Jacobs, R. Stahl, *Acta, Part A* **1998,** *54*, 893-901; c) J. Kloprogge, L*.* Hickey, R. Frost, *J. Raman Spectrosc.* **2004**, *35*, 967-974

[3] a) K. Zhu, T. Wu, K. Huang, *Chem. Mater.* **2021**, *33*, 4089-4098; b) Y. Kim, Y. Park, M. Kim, J. Lee, K. J. Kim, J. W. Choi, *Nat. Commun.* **2022**, *13*, 2371; c) W. Zhong, Z. Shen, J. Mao, S. Zhang, H. Cheng, K. Yoonseob, Y. Lu, *Energy Environ. Sci.* **2024**, *17*, 2059-2068.

[4] M. Yan, P. He, Y. Chen, S. Wang, Q. Wei, K. Zhao, X. Xu, Q. An, Y. Shuang, Y. Shao, K T. Mueller, L. Mai, J. Liu, J. Yang, *Adv. Mater.* **2018**, *30*, 1703725.

[5] Y. Lu, T. Zhu, W. Bergh, M. Stefik, K. Huang, *Angew. Chem. Int. Ed*. **2020**, *59*, 17004–17011.
